# Supplementary material for: Prevalence of atrial fibrillation in Northern Sri Lanka: a study protocol for a cross-sectional household survey
Source: BMJ Open. 2022 Nov 23;12(11):e056480. doi: 10.1136/bmjopen-2021-056480 (PMC9685242; doi:10.1136/bmjopen-2021-056480)
Supplement: Author's manuscript [file bmjopen-2021-056480.draft_revisions.pdf]

BMJ Open

BMJ Open is committed to open peer review. As part of this commitment we make the peer review history of every article we publish publicly available.

When an article is published we post the peer reviewers' comments and the authors' responses online. We also post the versions of the paper that were used during peer review. These are the versions that the peer review comments apply to.

The versions of the paper that follow are the versions that were submitted during the peer review process. They are not the versions of record or the final published versions. They should not be cited or distributed as the published version of this manuscript.

BMJ Open is an open access journal and the full, final, typeset and author-corrected version of record of the manuscript is available on our site with no access controls, subscription charges or pay-per-view fees (http://bmjopen.bmj.com).

If you have any questions on BMJ Open's open peer review process please email info.bmjopen@bmj.com

BMJ Open

Prevalence of atrial fibrillation in Northern Sri Lanka: A study protocol for a cross-sectional household survey

Journal:	*BMJ Open*
Manuscript ID	bmjopen-2021-056480
Article Type:	Protocol
Date Submitted by the Author:	18-Aug-2021

Complete List of Authors:	Kanesamoorthy, Shribavan; University of Jaffna Faculty of Medicine, Department of Community & Family Medicine<br>Sheron, Vethanayagam Antony; University of Jaffna Faculty of Medicine, Department of Community & Family Medicine<br>Uruthirakumar, Powsiga; University of Jaffna Faculty of Medicine, Department of Community & Family Medicine<br>Kodippily, Chamira; National Intensive Care Surveillance-Mahidol Oxford Tropical Medicine Research Unit<br>Kumarendran, Balachandran; University of Jaffna Faculty of Medicine, Department of Community & Family Medicine<br>Gooden, Tiffany; University of Birmingham College of Medical and Dental Sciences, Department of Public Health and Epidemiology<br>Thomas, G. Neil; University of Birmingham College of Medical and Dental Sciences, Department of Public Health and Epidemiology<br>Nirantharakumar, Krishnarajah; University of Birmingham College of Medical and Dental Sciences, Department of Public Health and Epidemiology<br>Lip, Gregory; University of Birmingham College of Medical and Dental Sciences, Department of Public Health and Epidemiology; University of Liverpool, Liverpool Centre for Cardiovascular Science<br>Guruparan, Mahesan; Jaffna Teaching Hospital<br>Haniffa, Rashan; National Intensive Care Surveillance-Mahidol Oxford Tropical Medicine Research Unit<br>Surenthirakumaran, Rajendra; University of Jaffna Faculty of Medicine, Department of Community & Family Medicine<br>Beane, Abi; National Intensive Care Surveillance-Mahidol Oxford Tropical Medicine Research Unit<br>Subaschandran, Kumaran; University of Jaffna Faculty of Medicine, Department of Community & Family Medicine

Keywords:	Cardiac Epidemiology < CARDIOLOGY, Telemedicine < BIOTECHNOLOGY & BIOINFORMATICS, Adult cardiology < CARDIOLOGY, Cardiology < INTERNAL MEDICINE, STATISTICS & RESEARCH METHODS, PREVENTIVE MEDICINE

For peer review only - http://bmjopen.bmj.com/site/about/guidelines.xhtml

Prevalence of atrial fibrillation in Northern Sri Lanka: A study protocol for a cross-sectional household survey

Shribavan Kanesamoorthy[1], Vethanayagam Antony Sheron[1], Powsiga Uruthirakumar[1], Chamira Kodippily[2], Balachandran Kumarendran[1], Tiffany E Gooden[3], G Neil Thomas[3*], Krishnarajah Nirantharakumar[3], Gregory YH Lip[3,4*], Mahesan Guruparan[5], Rashan Haniffa[2], Rajendra Surenthirakumaran[1*□], Abi Beane[2*], Kumaran Subaschandraen[1*□] on behalf of the NIHR Global Health Research Group on Atrial Fibrillation Management^

1. Department of Community & Family Medicine, Faculty of Medicine, University of Jaffna, Kokuvil, Sri Lanka

2. National Intensive Care Surveillance-Mahidol Oxford Tropical Medicine Research Unit, Jaffna, Sri Lanka

3. Institute of Applied Health Research, University of Birmingham, Birmingham, United Kingdom

4. Liverpool Centre for Cardiovascular Science, University of Liverpool, Liverpool, United Kingdom

5. Handy Memorial Cardiology Unit, Jaffna Teaching Hospital, Jaffna, Sri Lanka

* Profs Lip and Thomas are co-Directors of the NIHR Global Health Research Group on Atrial Fibrillation Management, and are joint senior authors with Drs Surenthirakumaran, Beane and Subaschandraen.

□ Joint corresponding authors:
Kumaran Subaschandraen: s.kumaran3@gmail.com University of Jaffna, Aadiyapatham Road, Kokuvil, Jaffna, Sri Lanka

Rajendra Surenthirakumaran: surenthirakumaran@gmail.com University of Jaffna, Aadiyapatham Road, Kokuvil, Jaffna, Sri Lanka

^ Members of the NIHR Global Health Group on Atrial Fibrillation Management listed in alphabetical order: Ajini Arasalingam, Abi Beane, Isabela Bensenor, Peter Brocklehurst, Kar Keung Cheng, Itamar de Souza Santos, Wahbi El-Bouri, Mei Feng, Alessandra Goulart, Sheila Greenfield, Yutao Guo, Mahesan Guruparan, Gustavo Gusso, Tiffany E Gooden, Rashan Haniffa, Lindsey Humphreys, Kate Jolly, Sue Jowett, Balachandran Kumarendran, Emma Lancashire, Deirdre A Lane, Xuewen Li, Gregory Y.H. Lip (Co-PI), Yan-guang Li, Trudie Lobban, Paulo Lotufo, Semira Manseki-Holland, David J Moore, Krishnarajah Nirantharakumar, Rodrigo Olmos, Elisabete Paschoal, Paskaran Pirasanth, Uruthirakumar

For peer review only

Powsiga, Carla Romagnolli Quintino, Alena Shantsila, Vethanayagam Antony Sheron, Kanesamoorthy Shribavan, Isabelle Szmigin, Kumaran Subaschandren, Rajendra Surenthirakumaran, Meihui Tai, Bamini Thavarajah, G. Neil Thomas (Co-PI) Ana Carolina Pontes Varella, Hao Wang, Jingya Wang, Hui Zhang, Jiaoyue Zhong.

Word count: 3053

Keywords: atrial fibrillation, low- and middle-income countries, epidemiological studies, community-based survey, screening programme, noncommunicable diseases

For peer review only

ABSTRACT

Introduction: Atrial fibrillation (AF) is the most common arrhythmia globally. It is associated with a 5-fold risk in stroke, but early diagnosis and effective treatment can reduce this risk. AF is often underdiagnosed, particularly in low- and middle-income countries (LMICs) where screening for AF is not always feasible or considered common practice in primary care settings. Epidemiological data on AF in LMICs is often incomplete particularly in vulnerable populations. This LMIC research collaborative aims to identify the prevalence of AF in the Northern Sri Lankan community.

Methods and analysis: A cross-sectional household survey piloted and codesigned through a series of community engagement events will be administered in all five districts in Northern Province, Sri Lanka. A multistage cluster sampling approach will be used starting at district level, then the Divisional Secretariats followed by Grama Niladhari divisions. Twenty households will be selected from each cluster. The study aims to recruit 10,000 participants aged 50 years or older, one participant per household. Demographic and socioeconomic characteristics, wellbeing and lifestyle and anthropometric measurements will be collected using a digital data platform (REDCap) by trained data collectors. Participants will be screened for AF using a fingertip single lead ECG via a smartphone application (AliveCor) with rhythm strips reviewed by a consultant cardiologist. Prevalence of AF and risk factors will be established at province and district-levels. Adjusted odds ratios and population attributable fractions for AF risk factors will be determined.

Ethics and dissemination: This study was approved by the Ethics Review Committee of Faculty of Medicine at University of Jaffna. Written informed consent will be obtained from all participants. Findings will be disseminated through publication in a peer-reviewed journal and presentations at conferences. The findings will enable early treatment for new AF diagnoses and inform interventions to improve community-based management of AF in LMICs.

For peer review only

STRENGTHS AND LIMITATIONS OF THIS STUDY

- This study utilises mHealth technology that has the ability to quickly and efficiently identify atrial fibrillation (AF) within resource-limited settings.
- The use of a near-real time data collection tool and analytical dashboard will enable the ability to identify and rectify any data collection issues as the study progresses.
- Conducting the study in this LMIC setting will inform future noncommunicable screening policies to extend the evidence base beyond high-income settings.
- Whilst data pertaining to a variety of risk factors will be collected, the temporal relationship between risk factors and AF will not be attained.
- Given the cross-sectional study design, incidence of AF and other noncommunicable diseases will not be feasible.

peer review only

INTRODUCTION

Atrial fibrillation (AF) is the commonest cardiac rhythm disorder globally.[1] AF prevalence is increasing worldwide with age, male sex, hypertension, diabetes and heart disease as major risk factors.[2] AF is associated with a 5-fold increase in stroke and over 25% of strokes are due to AF.[3] Early identification and effective management of AF can prevent strokes and contribute to improved management of cardiovascular health.[3-5] Stroke caused by AF is largely preventable with appropriate use of oral anticoagulant (OAC) therapy, a therapy which is readily available in health systems internationally.[5] However, an estimated 33% of people with AF are asymptomatic, meaning many people with AF remain undiagnosed until they present with life threatening complications.[6]

Screening for AF in people 65 years or older is now recommended by some clinical practice guidelines and is common place in general practice in upper-middle and high-income countries.[7, 8] Such population screening has in part been made possible by the successful development and scale up of smartphone technologies, which enable rapid point of care diagnosis through opportunistic pulse palpation or ECG rhythm strip.[9, 10]

Such practice remains limited in low- and middle-income countries (LMICs), where cardiovascular diseases in the younger populations are rapidly becoming the leading cause of mortality.[11] Additionally, complications associated with noncommunicable diseases are attributed with worse outcomes and greater morbidity for people living in LMICs when compared to high-income settings.[12] Despite this disparity in diagnosis, management and outcomes, there is limited high quality epidemiological data on AF in LMICs, specifically in South Asia.[2, 13] Screening programmes in the regions are hindered by poor recruitment, often as a result of poor community engagement and low levels of noncommunicable disease-related health literacy. Where programmes do exist, vulnerable groups are often underrepresented and missing or incomplete data inhibits meaningful interpretation of data.[14] Epidemiology researchers are increasingly looking to social science and implementation strategies to engage communities in research design to ensure they are included in such programmes. Particularly in LMICs, it is imperative to overcome these known barriers to study implementation and ensure that study findings are not only disseminated back to communities, but are both acceptable to and utilised by healthcare providers.

For peer review only - http://bmjopen.bmj.com/site/about/guidelines.xhtml

This study aims to implement an AF screening programme using smartphone technology, feasible for delivery at scale in resource constrained LMIC settings. Specifically, we aim to determine the prevalence of AF and other noncommunicable diseases in Northern Province, Sri Lanka and to evaluate the feasibility and acceptability of smartphone enabled single lead electrocardiogram (ECG) for the purpose of diagnosing AF.

METHODS AND ANALYSIS

Research design

This cross-sectional study combines a household questionnaire and an onsite assessment of the presence of AF using a single lead ECG reader on a digital mobile health (mHealth) platform. The study commenced in June 2020 and is expected to be complete by March 2022.

Study setting

This study is being conducted across all five districts in the Northern Province of Sri Lanka: Jaffna, Kilinochchi, Mannar, Mullaitivu and Vavuniya. Each district is divided into administrative units called Divisional Secretariats (DS). Each DS is further divided into many Grama Niladhari (GN) divisions. Each GN division is identified by a unique number. Northern Province is located just 22 miles (35 km) southeast of India and has approximately 1.3 million permanent residents.[15] Its climate is typically tropical, but with a hot dry season from February to September. Whilst the Northern Province was heavily impacted by 30 years of conflict, the province has recently opened to the global market and its share of Provincial Gross Domestic Product is increasing.[16]

Sampling and sample size

Individuals aged 50 years or above and fluent in the Tamil language will be considered eligible to participate. Participants suffering from terminal illnesses, any one in need of immediate hospital admission or those who are currently an inpatient in a hospital setting will be excluded.

This study will use a multistage cluster sampling approach. The multistage process will start at the district level, then to the DS level followed by the GN level. GN divisions will then be divided into clusters based on population size and from these clusters, a single cluster will be selected at random. Each cluster will consist of 20 households, one participant per household.

For peer review only

We will first randomly select an index house within each cluster and from the index house, 20 households on the right side will be selected. In households where more than one eligible person lives, the person with a birthday closest to the date of visit will be selected.

For the sample size calculation, the prevalence of AF in Sri Lanka was estimated to be 1% based on previous evidence from Asian populations.[2, 17] The sample size was calculated using standard published methods. We increased our sample size by 10% to account for non-participation,[18] leading to a minimum sample size of 10,000 participants required. The sampling frame for the study and population distributions are based on the 2012 population census.[19]

Pilot study

The data collection tool was piloted in 100 households located in a GN division that will not be included within the main study. The pilot study comprised participants from varying socioeconomic backgrounds and education levels. Participants were asked to give their perspectives on acceptability of participating in the study including the duration of the interview, ease of understanding the interviewer administered questionnaire and their experience of using the fingertip rhythm assessment ECG application. Additionally, data collectors fed back on the challenges they experienced in collecting the data and reported on their perception of participants' willingness to participate and recall, internal consistency reliability and interrater reliability of the questionnaire. During the piloting of the questionnaire, participants' questions and concerns were noted and reported through the electronic data collection tool used; these were reviewed daily by the principal and co-investigators. Recurring themes regarding concerns over the reasons for conducting the survey and potentially being disadvantaged if they were found to have AF prompted a review of participant information leaflets and further training for the data collection team.

For peer review only

Data collection

The household survey comprises five sections: i) demographics and socioeconomic profile; ii) current and past health conditions; iii) wellbeing and lifestyle; iv) anthropometric measurements and; v) screening for AF. The demographic and socioeconomic profile will be collected using the Sri Lanka Demographic and Health Survey 2016 questionnaire which includes education levels and occupation.[20] Current medical conditions and medical history of comorbid diseases will be collected using the Charlson Comorbidity Index [21] along with the recording of any current medications prescribed by physicians or indigenous healers. To

assist participant's recall of their medical history, the data collection team will advise them to review their 'clinic books' which is a paper notebook provided by the health system and commonly kept by patients who see clinicians for long standing conditions in which clinic appointments, clinical assessments, investigations and medications are often detailed. Wellbeing and lifestyle assessment will be collected using the following validated instruments: General Anxiety Disorder scale (GAD-2),[22] Patient Health Questionnaire-2 (PHQ-2),[23] International Physical Activity Questionnaire - Short Form (IPAQ-SF),[24] Audit C for alcohol use,[25] WHO STEPS instrument [26] for assessing tobacco use, Berlin Questionnaire for sleep quality,[27] EQ-5D-5L for quality of life,[28] and the Fried Frailty Index [29]. Diet will also be assessed by asking questions on consumption levels of relevant food groups.[30] Participants will be asked to describe their utilisation of healthcare services in the preceding year, including with public and private services and indigenous healers. Anthropometric measurements will include the recording of each participant's weight, height, waist and hip circumference along with their blood pressure (measured by data collectors).

Participants will have their heart rhythm recorded using fingertip electrodes which activate a bipolar single lead (lead 1) ECG recording through a smartphone application (AliveCor) that uses an automated algorithm to interpret the cardiac rhythm and identify AF.[31] AliveCor is a validated tool used internationally and is FDA approved.[32, 33] Most studies have found the sensitivity and specificity of AliveCor to be 90% or above.[32] The application logs the participants anonymously, assigning a unique ID to each participant and stores a report of the rhythm detected. The participant's unique ID is entered into the data collection tool to enable linkage of the participant's heart rhythm results and their questionnaire data. Reports of the single lead recording will be downloaded and reviewed by a consultant cardiologist off-site.

Digital data collection tools that had been developed and successfully deployed in Sri Lanka were adapted for use in this study.[34] We improved data quality and standardised terminology for diagnosis and comorbidities and included automatic data-logic checks for numerical values to reduce data entry errors. The final tool, utilising the REDCap (Research Electronic Data Capture) [35] software, includes satellite mapping and inbuilt date and time stamps, and supports study coordinators with monitoring data collection and feedback. Data collected is automatically transferred to analytical dashboards (figure 1); thus, the study research team has in near-real time access to study recruitment, data quality, reasons for failure to consent and feasibility of study components. The digital tool is accessible via a

For peer review only

mobile device and includes offline functionality, enabling the ability to overcome any issues with mobile data coverage.

The questionnaire and study tools were designed in English and then translated to Tamil by two independent translators. These translations were then reviewed by a working group of bilingual stakeholders from a range of academic, education and professional backgrounds to ensure that the translation and phrasing of the questionnaire was appropriate for monolingual participants. The refined Tamil version of the questionnaire was then back translated to English and reviewed for integrity and retention of information by the study team.

For every cluster GN, a community volunteer will facilitate the data collection by introducing the data collectors to participants. Each data collection team will consist of a trained data collector and the community volunteer. Data collectors will be trained by researchers and community physicians from the University of Jaffna in study participation, consent process, administering the questionnaire and data entry using the digital data collection tool. Data collectors will also be trained in how to administer AliveCor for detecting AF. Trained data collectors will provide information to the participant regarding the study, and obtain written informed consent.

Statistical methods

A direct standardisation technique will be used to obtain province-level prevalence data from the district level results. The proportion of population exposed to known risk factors of AF will be calculated. Prevalence of risk factors in participants with established, without and with newly diagnosed AF will be determined. Multivariate logistic regressions will be used to calculate adjusted odd ratios for AF risk factors. Population attributable fractions will be calculated for risk factors that were included in the logistic regression model. Statistical analysis would be carried out using SPSS Version 23 (IBM Corp. 2015; Armonk, NY). Raw data will be reported along with scores from assessment tools (e.g GAD-2 and PHQ-2), derived using published definitions. All calculations will be published with open access, ensuring reproducibility of study findings.

Study monitoring

Data quality (completeness, accuracy, logic) will be reviewed daily by the study's data quality coordinators from a central monitoring platform. The study will adhere to the FAIR guiding principles of data management and sharing.[36] In-built dashboards will provide

For peer review only

accrued metadata on screening for AF, eligibility, participant recruitment, data quality and prevalence of AF and other noncommunicable diseases in the study population.[34, 37] Weekly debriefs between the study team and data collectors will be used to discuss progress and address any challenges arising. In addition, source data will be verified in 1% of questionnaires at random each month where research fellows will join the data collectors and observe study conduct and identify any potential biases in data collection which may impact interrater reliability.

Data storage and security

Data will be stored on institutional network drives of the Information and Communication Technology Agency Sri Lanka, adhering to national data storage requirements.[38] Access to records and data will be limited to study personnel. All participant data will be anonymised by removing any identifiable data from the data collected in field.[39] The data may be linked using a serial number.

Patient and public involvement

Community engagement workshops with key stakeholders were conducted by the study team. Medical Officers of Health (MOHs) and Public Health Midwives (PHMs) within each GN division were asked to participate and aid in the community engagement activities. Stakeholders included patient representatives and primary health care physicians, community health workers and specialist physicians working in the Northern Province. Stakeholders were asked to give their opinions on the research objectives and their perceptions of potential challenges of conducting large scale epidemiological studies in the province. This included how to achieve participant engagement from marginalised communities, improve health literacy and health seeking behaviours and how to optimise data quality and impact population health policy.[40] These insights were used to inform the selection of study tools for exploring health seeking behaviours, lifestyle assessment and participant information. Similar workshops are planned at three-month intervals during participant enrollment, data collection and analysis, whereby emerging enablers and barriers to the study will be disseminated to stakeholders. Thus, we will create a dialog whereby perceptions of current services can be evaluated and priorities for improvement be identified and agreed with stakeholders throughout the study. Participants from the stakeholder group will be asked to join a smaller working group who will guide the development of health education materials pertaining to lifestyle, general wellbeing and AF specific management. They will provide

For peer review only

feedback on the materials, their acceptability, potential barriers to the adoption of new knowledge by the Northern Province community and codesign future implementation methods of the materials.

COVID-19 pandemic

Whilst the pandemic was emerging in Europe and the US, COVID-19 cases were not yet present in Sri Lanka. We utilised this opportunity to discuss perceptions of the disease at the community engagement workshops, assess risk and adapt data collection procedures to reduce risk of transmission. Preventive measures such as hand washing techniques, maintaining social distancing and wearing face masks were explained to data collectors. Hand sanitiser was distributed to all data collectors and they were instructed to disinfect their hands frequently.

ETHICS AND DISSEMINATION

The study received approval from the Ethics Review Committee of Faculty of Medicine, University of Jaffna (J/ERC/19/103/NDR/0208). Written informed consent will be obtained from all participants. A helpline for participants and family members and caretakers of the participants will be set up for any questions or concerns throughout and beyond the study period.

Any participant that is newly diagnosed with AF through this study will be given a clinical appointment to see the study consultant cardiologist. Results of the study will be made available to participants and/or their caregivers, as appropriate. The findings will be disseminated through abstract presentations at national and international conferences and a manuscript publication in a peer-reviewed journal.

DISCUSSION

This study will be the first of its kind in Sri Lanka and one of few community-based AF screening studies conducted in South Asia.[2] Our findings will determine the prevalence of AF within the Sri Lankan community and enable early and effective treatment for those newly diagnosed with AF. Hospital data in Sri Lanka and other LMICs are often limited, is of poor-quality and typically does not represent the true burden of conditions due to various reasons (healthcare seeking behaviours, lack of diagnostic equipment in primary care, inefficient referral systems, patients lost to follow-up etc.).[41-43] This study will also

For peer review only

highlight key enablers and barriers of implementing a population-based screening programme for AF in an LMIC setting, facilitating other LMICs with the means to adapt and implement similar programmes. Dissemination of these findings will provide evidence for policy makers and healthcare professionals of the true burden of AF within the communities they serve, enabling a discourse on how to develop effective care and management to reduce incident AF and improve timely diagnosis of existing AF.

Further to the study aims, recruitment of the 10,000 participants will form a distinct cohort of community members that can be utilised in future studies. All participants that consent to being included within the cohort will be contacted for future trials and surveys regarding AF and other noncommunicable diseases. To our knowledge, no AF community-based cohort currently exists in South East Asia. This study along with future studies carried out within this cohort will enhance the epidemiological evidence on noncommunicable diseases in Sri Lanka. Subsequently, interventions can be developed and tested for improving the health and wellbeing of LMIC occupants, including those most vulnerable and disadvantaged.

The strengths of this study include the use of mHealth technology for screening for and diagnosing AF in an LMIC setting. In high-income countries the tool has been shown to be easy to use, cost-effective and yields a high accuracy rate.[32] Success of using this tool within the study will indicate the feasibility of using it in other resource-limited settings (including post-war settings) either in the community or in primary care settings that lack appropriate equipment. Conducting this study in an LMIC setting will provide important learnings for future screening programmes in LMICs, extending the current evidence beyond high-income countries. The use of analytical dashboards is another strength of the study. In near-real time, data is able to be reviewed and assessed by the study team both nationally and internationally for any quality issues, enabling immediate rectification and ongoing monitoring. The dashboards also allow for a unique opportunity to review the study findings as data collection progresses. There are however some limitations to be mentioned. Due to the cross-sectional design of this study, only prevalence of AF will be feasible; therefore, AF incidence in the community will remain unknown. Another limitation due to the study design is the inability to infer on the causal relationship between risk factors and AF. Though, the collection of a wide range of demographic and lifestyle data will allow for determination of which risk factors are associated with an existing and new AF diagnosis.

For peer review only

In conclusion, our study will identify the prevalence of AF in the Sri Lankan general population. Further to this, our findings will enable learnings for screening programmes for other LMICs and provide evidence on the feasibility of using a mHealth tool for diagnosing AF in a resource-limited setting. Such learnings have the potential to improve early diagnosis and prevention of AF in LMICs, which will increasingly become a global health priority.

Figure 1. Analytical online dashboard. A breakdown of demographics, lifestyle and wellbeing measures for participants recruited and consented thus far.

Acknowledgements: The authors would like to thank the MOHs, PHMs, data collectors and volunteers for their dedication and invaluable assistance with the pilot study and with recruitment thus far.

Author's contribution: BK, GNT, KN, GYHL, MG, RS and KS conceptualised and designed the study. All authors contributed to the piloting and implementation of the study. SK, TEG and AB drafted the manuscript. All authors reviewed and approved the final manuscript for submission.

Funding: This work was supported by the National Institute for Health Research (project reference 17/63/121, NIHR Global Health Research Group on Atrial Fibrillation Management). The funder had no role in the design of the study or the writing of this protocol.

Competing interests: GYHL is a consultant and speaker for BMS/Pfizer, Boehringer Ingelheim and Daiichi-Sankyo; no fees are received personally. No other authors have any competing interests to declare.

For peer review only

REFERENCES

1. Chugh SS, Roth GA, Gillum RF, et al. Global burden of atrial fibrillation in developed and developing nations. *Glob Heart*. 2014;9:113-9.

2. Zulkifly H, Lip GY, Lane DA. Epidemiology of atrial fibrillation. *Int J Clin Pract*. 2018;72:e13070.

3. Björck S, Palaszewski B, Friberg L, et al. Atrial fibrillation, stroke risk, and warfarin therapy revisited: a population-based study. *Stroke*. 2013;44:3103-8.

4. Katsanos AH, Kamel H, Healey JS, et al. Stroke prevention in atrial fibrillation: looking forward. *Circulation*. 2020;142:2371-88.

5. Chao T-F, Nedeljkovic MA, Lip GY, et al. Stroke prevention in atrial fibrillation: comparison of recent international guidelines. *Eur Heart J Suppl*. 2020;22:O53-O60.

6. Xiong Q, Proietti M, Senoo K, et al. Asymptomatic versus symptomatic atrial fibrillation: a systematic review of age/gender differences and cardiovascular outcomes. *Int J Cardiol*. 2015;191:172-7.

7. Freedman B, Camm J, Calkins H, et al. Screening for atrial fibrillation: a report of the AF-SCREEN International Collaboration. *Circulation*. 2017;135:1851-67.

8. Hindricks G, Potpara T, Dagres N, et al. 2020 ESC Guidelines for the diagnosis and management of atrial fibrillation developed in collaboration with the European Association for Cardio-Thoracic Surgery (EACTS) The Task Force for the diagnosis and management of atrial fibrillation of the European Society of Cardiology (ESC) Developed with the special contribution of the European Heart Rhythm Association (EHRA) of the ESC. *Eur Heart J*. 2021;42:373-498.

9. Lee J, Reyes BA, McManus DD, et al. Atrial fibrillation detection using an iPhone 4S. *IEEE Trans Biomed Eng*. 2012;60:203-6.

10. Orchard J, Lowres N, Freedman SB, et al. Screening for atrial fibrillation during influenza vaccinations by primary care nurses using a smartphone electrocardiograph (iECG): a feasibility study. *Eur J Prev Cardiol*. 2016;23:13-20.

11. Bowry AD, Lewey J, Dugani SB, et al. The burden of cardiovascular disease in low-and middle-income countries: epidemiology and management. *Can J Cardiol*. 2015;31:1151-9.

12. Rosengren A, Smyth A, Rangarajan S, et al. Socioeconomic status and risk of cardiovascular disease in 20 low-income, middle-income, and high-income countries: the Prospective Urban Rural Epidemiologic (PURE) study. *Lancet Glob Health*. 2019;7:e748-e60.

13. Bai Y, Wang Y-L, Shantsila A, et al. The global burden of atrial fibrillation and stroke: a systematic review of the clinical epidemiology of atrial fibrillation in Asia. *Chest*. 2017;152:810-20.

For peer review only

14. Santos IS, Goulart AC, Olmos RD, et al. Atrial fibrillation in low-and middle-income countries: a narrative review. *Eur Heart J Suppl*. 2020;22:O61-O77.

15. Northern Provincial Council. Vital Statistics 2020. Jaffna, Sri Lanka: Office of the Deputy Chief Secretary; 2020. https://np.gov.lk/vital-statistics-2020/ (accessed 27 July 2021).

16. Central Bank of Sri Lanka. Provincial Gross Domestic Product - 2019. Colombo, Sri Lanka: Statistics Department; 2020. https://www.cbsl.gov.lk/en/news/provincial-gross-domestic-product-2019# (accessed 27 July 2021).

17. O'Neill J, Tayebjee MH. Why are South Asians seemingly protected against the development of atrial fibrillation? A review of current evidence. *Trends Cardiovasc Med*. 2017;27:249-57.

18. Martínez-Mesa J, González-Chica DA, Bastos JL, et al. Sample size: how many participants do I need in my research? *An Bras Dermatol*. 2014;89:609-15.

19. Department of Census and Statistics Sri Lanka. Census of Population and Housing Sri Lanka 2012. Sri Lanka: Department of Census and Statistics Sri Lanka; 2012. http://www.statistics.gov.lk/Population/StaticalInformation/CPH2011 (accessed 27 July 2021).

20. Department of Census and Statistics Sri Lanka. Demographic and Health Survey 2016 Sri Lanka: Department of Census and Statistics Sri Lanka; 2016. http://www.statistics.gov.lk/Health/StaticalInformation (accessed 27 July 2021).

21. Austin SR, Wong Y-N, Uzzo RG, et al. Why summary comorbidity measures such as the Charlson comorbidity index and Elixhauser score work. *Med Care*. 2015;53:e65.

22. Kroenke K, Spitzer RL, Williams JB, et al. Anxiety disorders in primary care: prevalence, impairment, comorbidity, and detection. *Ann Intern Med*. 2007;146:317-25.

23. Arroll B, Goodyear-Smith F, Crengle S, et al. Validation of PHQ-2 and PHQ-9 to screen for major depression in the primary care population. *Ann Fam Med*. 2010;8:348-53.

24. Lee PH, Macfarlane DJ, Lam TH, et al. Validity of the international physical activity questionnaire short form (IPAQ-SF): A systematic review. *Int J Behav Nutr Phys Act*. 2011;8:1-11.

25. Bush K, Kivlahan DR, McDonell MB, et al. The AUDIT alcohol consumption questions (AUDIT-C): an effective brief screening test for problem drinking. *Arch Intern Med*. 1998;158:1789-95.

26. World Health Organization. WHO STEPS surveillance manual: the WHO STEPwise approach to chronic disease risk factor surveillance. Geneva: World Health Organization; 2005. https://apps.who.int/iris/handle/10665/43376 (accessed 27 July 2021).

27. Netzer NC, Stoohs RA, Netzer CM, et al. Using the Berlin Questionnaire to identify patients at risk for the sleep apnea syndrome. *Ann Intern Med*. 1999;131:485-91.

For peer review only

28. Herdman M, Gudex C, Lloyd A, et al. Development and preliminary testing of the new five-level version of EQ-5D (EQ-5D-5L). *Qual Life Res*. 2011;20:1727-36.

29. Revenig LM, Canter DJ, Kim S, et al. Report of a simplified frailty score predictive of short-term postoperative morbidity and mortality. *J Am Coll Surg*. 2015;220:904-11. e1.

30. Schooling CM, Ho SY, Leung GM, et al. Diet synergies and mortality—a population-based case–control study of 32 462 Hong Kong Chinese older adults. *Int J Epidemiol*. 2006;35:418-26.

31. Galloway CD, Albert DE, Freedman SB. iPhone ECG application for community screening to detect silent atrial fibrillation: a novel technology to prevent stroke. *Int J Cardiol*. 2013;165:193-4.

32. Hall A, Mitchell ARJ, Wood L, et al. Effectiveness of a single lead AliveCor electrocardiogram application for the screening of atrial fibrillation: a systematic review. *Medicine (Baltimore)*. 2020;99.

33. Cheung CC, Krahn AD, Andrade JG. The emerging role of wearable technologies in detection of arrhythmia. *Can J Cardiol*. 2018;34:1083-7.

34. Beane A, De Silva AP, Athapattu PL, et al. Addressing the information deficit in global health: lessons from a digital acute care platform in Sri Lanka. *BMJ Glob Health*. 2019;4:e001134.

35. REDCap. REDCap: Research Electronic Data Capture Nashville, Tennessee: Vanderbilt University. https://projectredcap.org/ (accessed 12 August 2021).

36. Wilkinson MD, Dumontier M, Aalbersberg IJ, et al. The FAIR Guiding Principles for scientific data management and stewardship. *Sci Data*. 2016;3:1-9.

37. CRIT Care Asia, Hashmi M, Beane A, et al. Leveraging a cloud-based critical care registry for COVID-19 pandemic surveillance and research in low-and middle-income countries. *JMIR Public Health Surveill*. 2020;6:e21939.

38. Information and Communication Technology Agency of Sri Lanka. Security Sri Lanka: Information and Communication Technology Agency of Sri Lanka; [Available from: https://www.icta.lk/security/ (accessed 27 July 2021).

39. Centers for Disease Control Prevention. HIPAA privacy rule and public health. Guidance from CDC and the US Department of Health and Human Services. *MMWR Morb Mortal Wkly Rep*. 2003;52:1-17, 9.

40. Cook N, Siddiqi N, Twiddy M, et al. Patient and public involvement in health research in low and middle-income countries: a systematic review. *BMJ Open*. 2019;9:e026514.

41. Arsenault C, Kim MK, Aryal A, et al. Hospital-provision of essential primary care in 56 countries: determinants and quality. *Bull World Health Organ*. 2020;98:735.

42. Chow CK, Nguyen TN, Marschner S, et al. Availability and affordability of medicines and cardiovascular outcomes in 21 high-income, middle-income and low-income countries. *BMJ Glob Health*. 2020;5:e002640.

For peer review only

43. Lowres N, Giskes K, Hespe C, et al. Reducing stroke risk in atrial fibrillation: adherence to guidelines has improved, but patient persistence with anticoagulant therapy remains suboptimal. *Korean Circ J*. 2019;49:883-907.

For peer review only

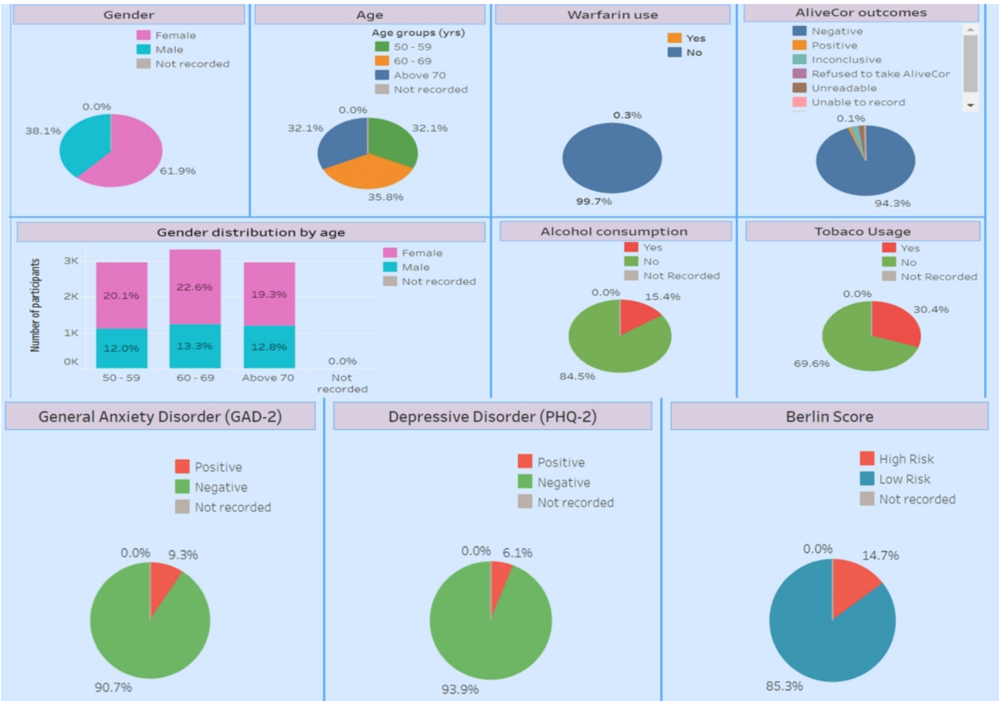

Analytical online dashboard. A breakdown of demographics, lifestyle and wellbeing measures for participants recruited and consented thus far.

Prevalence of atrial fibrillation in Northern Sri Lanka: A study protocol for a cross-sectional household survey

Journal:	*BMJ Open*
Manuscript ID	bmjopen-2021-056480.R1
Article Type:	Protocol
Date Submitted by the Author:	11-Aug-2022

Complete List of Authors:	Kanesamoorthy, Shribavan; University of Jaffna Faculty of Medicine, Department of Community & Family Medicine<br>Sheron, Vethanayagam Antony; University of Jaffna Faculty of Medicine, Department of Community & Family Medicine<br>Uruthirakumar, Powsiga; University of Jaffna Faculty of Medicine, Department of Community & Family Medicine<br>Kodippily, Chamira; National Intensive Care Surveillance-Mahidol Oxford Tropical Medicine Research Unit<br>Kumarendran, Balachandran; University of Jaffna Faculty of Medicine, Department of Community & Family Medicine<br>Gooden, Tiffany; University of Birmingham College of Medical and Dental Sciences, Department of Public Health and Epidemiology<br>Thomas, G. Neil; University of Birmingham College of Medical and Dental Sciences, Department of Public Health and Epidemiology<br>Nirantharakumar, Krishnarajah; University of Birmingham College of Medical and Dental Sciences, Department of Public Health and Epidemiology<br>Lip, Gregory; University of Birmingham College of Medical and Dental Sciences, Department of Public Health and Epidemiology; University of Liverpool, Liverpool Centre for Cardiovascular Science<br>Guruparan, Mahesan; Jaffna Teaching Hospital<br>Haniffa, Rashan; The University of Edinburgh Centre for Inflammation Research<br>Surenthirakumaran, Rajendra; University of Jaffna Faculty of Medicine, Department of Community & Family Medicine<br>Beane, Abi; The University of Edinburgh Centre for Inflammation Research<br>Subaschandran, Kumaran; University of Jaffna Faculty of Medicine, Department of Community & Family Medicine

**Primary Subject Heading**:	Diagnostics
Secondary Subject Heading:	Cardiovascular medicine, Epidemiology, Global health, Research methods
Keywords:	Cardiac Epidemiology < CARDIOLOGY, Telemedicine < BIOTECHNOLOGY & BIOINFORMATICS, Adult cardiology < CARDIOLOGY, Cardiology < INTERNAL MEDICINE, STATISTICS & RESEARCH METHODS, PREVENTIVE MEDICINE

Prevalence of atrial fibrillation in Northern Sri Lanka: A study protocol for a cross-sectional household survey

Shribavan Kanesamoorthy[1], Vethanayagam Antony Sheron[1], Powsiga Uruthirakumar[1], Chamira Kodippily[2], Balachandran Kumarendran[1], Tiffany E Gooden[3], G Neil Thomas[3*], Krishnarajah Nirantharakumar[3], Gregory YH Lip[3,4*], Mahesan Guruparan[5], Rashan Haniffa[2], Rajendra Surenthirakumaran[1*□], Abi Beane[2*], Kumaran Subaschandran[1*□] on behalf of the NIHR Global Health Research Group on Atrial Fibrillation Management^

1. Department of Community & Family Medicine, Faculty of Medicine, University of Jaffna, Kokuvil, Sri Lanka

2. National Intensive Care Surveillance-Mahidol Oxford Tropical Medicine Research Unit, Jaffna, Sri Lanka

3. Institute of Applied Health Research, University of Birmingham, Birmingham, United Kingdom

4. Liverpool Centre for Cardiovascular Science, University of Liverpool, Liverpool, United Kingdom

5. Handy Memorial Cardiology Unit, Jaffna Teaching Hospital, Jaffna, Sri Lanka

* Profs Lip and Thomas are co-Directors of the NIHR Global Health Research Group on Atrial Fibrillation Management, and are joint senior authors with Drs Surenthirakumaran, Beane and Subaschandran.

□ Joint corresponding authors:
Kumaran Subaschandran: s.kumaran3@gmail.com University of Jaffna, Aadiyapatham Road, Kokuvil, Jaffna, Sri Lanka

Rajendra Surenthirakumaran: surenthirakumaran@gmail.com University of Jaffna, Aadiyapatham Road, Kokuvil, Jaffna, Sri Lanka

^ Members of the NIHR Global Health Group on Atrial Fibrillation Management listed in alphabetical order: Ajini Arasalingam, Abi Beane, Isabela M Bensenor, Peter Brocklehurst, Kar Keung Cheng, Wahbi El-Bouri, Mei Feng, Alessandra C Goulart, Sheila Greenfield, Yutao Guo, Mahesan Guruparan, Gustavo Gusso, Tiffany E Gooden, Rashan Haniffa, Lindsey Humphreys, Kate Jolly, Sue Jowett, Chamira Kodippily, Balachandran Kumarendran, Emma Lancashire, Deirdre A Lane, Xuewen Li, Gregory Y.H. Lip (Co-PI), Yan-guang Li, Trudie Lobban, Paulo A Lotufo, Semira Manaseki-Holland, David Moore, Krishnarajah Nirantharakumar, Rodrigo D Olmos, Elisabete Paschoal, Paskaran Pirasanth,

For peer review only

Uruthirakumar Powsiga, Carla Romagnolli, Itamar S Santos, Alena Shantsila, Vethanayagan Antony Sheron, Kanesamoorthy Shribavan, Isabelle Szmigin, Kumaran Subaschandrn, Rajendra Surenthirakumaran, Meihui Tai, G. Neil Thomas (Co-PI), Timo Tolppa, Ana C Varella, Hao Wang, Jingya Wang, Hui Zhang, Jiaoyue Zhong.

Word count: 3384

Keywords: atrial fibrillation, low- and middle-income countries, epidemiological studies, community-based survey, screening programme, noncommunicable diseases

For peer review only

ABSTRACT

Introduction: Atrial fibrillation (AF) is the most common arrhythmia globally. It is associated with a 5-fold risk in stroke, but early diagnosis and effective treatment can reduce this risk. AF is often underdiagnosed, particularly in low- and middle-income countries (LMICs) where screening for AF is not always feasible or considered common practice in primary care settings. Epidemiological data on AF in LMICs is often incomplete particularly in vulnerable populations. This LMIC research collaborative aims to identify the prevalence of AF in the Northern Sri Lankan community.

Methods and analysis: A cross-sectional household survey piloted and codesigned through a series of community engagement events will be administered in all five districts in Northern Province, Sri Lanka. A multistage cluster sampling approach will be used starting at district level, then the Divisional Secretariats followed by Grama Niladhari divisions. Twenty households will be selected from each cluster. The study aims to recruit 10,000 participants aged 50 years or older, one participant per household. Demographic and socioeconomic characteristics, wellbeing and lifestyle and anthropometric measurements will be collected using a digital data platform (REDCap) by trained data collectors. Participants will be screened for AF using a fingertip single lead ECG via a smartphone application (AliveCor) with rhythm strips reviewed by a consultant cardiologist. Prevalence of AF and risk factors will be established at province and district-levels. Adjusted odds ratios and population attributable fractions for AF risk factors will be determined.

Ethics and dissemination: This study was approved by the Ethics Review Committee of Faculty of Medicine at University of Jaffna. Written informed consent will be obtained from all participants. Findings will be disseminated through publication in a peer-reviewed journal and presentations at conferences. The findings will enable early treatment for new AF diagnoses and inform interventions to improve community-based management of AF in LMICs.

For peer review only

STRENGTHS AND LIMITATIONS OF THIS STUDY

- This study utilises mHealth technology that has the ability to quickly and efficiently identify atrial fibrillation (AF) within resource-limited settings.
- The use of a near-real time data collection tool and analytical dashboard will enable the ability to identify and rectify any data collection issues as the study progresses.
- Conducting the study in this LMIC setting will inform future noncommunicable screening policies to extend the evidence base beyond high-income settings.
- Whilst data pertaining to a variety of risk factors will be collected, the temporal relationship between risk factors and AF will not be attained.
- Given the cross-sectional study design, incidence of AF and other noncommunicable diseases will not be feasible.

peer review only

INTRODUCTION

Atrial fibrillation (AF) is the commonest cardiac rhythm disorder globally.[1] AF prevalence is increasing worldwide with age, male sex, hypertension, diabetes and heart disease as major risk factors.[2] AF is associated with a 5-fold increase in stroke and over 25% of strokes are due to AF.[3] Early identification and effective management of AF can prevent strokes and contribute to improved management of cardiovascular health.[3-5] Stroke caused by AF is largely preventable with appropriate use of oral anticoagulant (OAC) therapy, a therapy which is readily available in health systems internationally.[5] However, an estimated 33% of people with AF are asymptomatic, meaning many people with AF remain undiagnosed until they present with life threatening complications.[6]

Screening for AF in people 65 years or older is now recommended by some clinical practice guidelines and is common place in general practice in upper-middle and high-income countries.[7,8] Such population screening has in part been made possible by the successful development and scale up of smartphone technologies, which enable rapid point of care diagnosis through opportunistic pulse palpation or ECG rhythm strip.[9,10]

Such practice remains limited in low- and middle-income countries (LMICs), where cardiovascular diseases in the younger populations are rapidly becoming the leading cause of mortality.[11] As a result, literature increasingly supports an age threshold of 50 years and above for AF screening.[12-14] Additionally, complications associated with noncommunicable diseases are attributed with worse outcomes and greater morbidity for people living in LMICs when compared to high-income settings.[15] Despite this disparity in diagnosis, management and outcomes, there is limited high quality epidemiological data on AF in LMICs, specifically in South Asia.[2,16] Screening programmes in the regions are hindered by poor recruitment, often as a result of poor community engagement and low levels of noncommunicable disease-related health literacy. Where programmes do exist, vulnerable groups are often underrepresented and missing or incomplete data inhibits meaningful interpretation of data.[17] Epidemiology researchers are increasingly looking to social science and implementation strategies to engage communities in research design to ensure they are included in such programmes. Particularly in LMICs, it is imperative to overcome these known barriers to study implementation and ensure that study findings are not only disseminated back to communities, but are both acceptable to and utilised by healthcare providers.

For peer review only - http://bmjopen.bmj.com/site/about/guidelines.xhtml

This study aims to implement an AF screening programme using smartphone technology, feasible for delivery at scale in resource constrained LMIC settings. Specifically, we aim to determine the prevalence of AF and associated risk factors in Northern Province, Sri Lanka and to evaluate the feasibility and acceptability of smartphone enabled single lead electrocardiogram (ECG) for the purpose of diagnosing AF.

METHODS AND ANALYSIS

Research design

This cross-sectional study combines a household questionnaire and an onsite assessment of the presence of AF using a single lead ECG reader on a digital mobile health (mHealth) platform. The study commenced in June 2020 and is expected to be complete by March 2022.

Study setting

This study is being conducted across all five districts in the Northern Province of Sri Lanka: Jaffna, Kilinochchi, Mannar, Mullaitivu and Vavuniya. Each district is divided into administrative units called Divisional Secretariats (DS). Each DS is further divided into many Grama Niladhari (GN) divisions. Each GN division is identified by a unique number. Northern Province is located just 22 miles (35 km) southeast of India and has approximately 1.3 million permanent residents.[18] Its climate is typically tropical, but with a hot dry season from February to September. Whilst the Northern Province was heavily impacted by 30 years of conflict, the province has recently opened to the global market and its share of Provincial Gross Domestic Product is increasing.[19] Publicly available healthcare including medical appointments, medications and medical procedures are free to all Sri Lankan citizens.

Sampling and sample size

Consistent with other LMIC studies,[12] individuals aged 50 years or above will be considered eligible; additionally, theymust be fluent in the Tamil language. Based on already published methods,[20] anyone suffering from terminal illnesses, in need of immediate hospital admission or currently an inpatient in a hospital setting will be excluded.

This study will use a multistage cluster sampling approach. The multistage process will start at the district level, then to the DS level followed by the GN level. GN divisions will then be divided into clusters based on population size and from these clusters, a single cluster will be

For peer review only

selected at random. Each cluster will consist of 20 households, one participant per household. We will first randomly select an index house within each cluster and from the index house, 20 households on the right side will be selected. In households where more than one eligible person lives, the person with a birthday closest to the date of visit will be selected. We used 2012 population census data to select the clusters and index house within each cluster.[19]

For the sample size calculation, the prevalence of AF in Sri Lanka was estimated to be 1% based on previous evidence.[2,21,22] We used a design effect of 2 to adjust for cluster sampling, and we used an alpha level of 5% and beta level of 20%. We increased our sample size by 10% to account for non-participation,[23] leading to a minimum sample size of 10,000 participants required.

Pilot study

The data collection tool was piloted in 100 households located in a GN division that will not be included within the main study. The pilot study comprised participants from varying socioeconomic backgrounds and education levels. Participants were asked to give their perspectives on acceptability of participating in the study including the duration of the interview, ease of understanding the interviewer administered questionnaire and their experience of using the fingertip rhythm assessment ECG application. Additionally, data collectors fed back on the challenges they experienced in collecting the data and reported on their perception of participants' willingness to participate and recall, internal consistency reliability and interrater reliability of the questionnaire. During the piloting of the questionnaire, participants' questions and concerns were noted and reported through the electronic data collection tool used; these were reviewed daily by the principal and co-investigators. Feedback following the pilot revealed i, data collector concerns regarding availability of and access to clinic books for relevant medical history, ii, participant concerns regarding future use of fingerprints, reasons for the survey and being disadvantaged if they were found to have AF, and iii, participants understanding of medical prescriptions. In response, we made the following adaptations to the study conduct protocol prior to commencing data collection: reporting of medical history will be reported as either being directly observed from clinic books or via participant recall; medications will be reported by categories for simplicity and; an update of participant information leaflets to insure the aim of the study, data security and what happens after the study is clear. Additionally, further training for the data collection team was provided on the management of personal information, the AliveCor device and how to respond to participant concerns.

For peer review only

Data collection

The household survey comprises five sections: i) demographics and socioeconomic profile; ii) current and past health conditions; iii) wellbeing and lifestyle; iv) anthropometric measurements and; v) screening for AF. The demographic and socioeconomic profile will be collected using the Sri Lanka Demographic and Health Survey 2016 questionnaire which includes education levels and occupation.[24] Current medical conditions and medical history of comorbid diseases will be collected using the Charlson Comorbidity Index [25] along with the recording of any current medications prescribed by physicians or indigenous healers. To assist participant's recall of their medical history, the data collection team will advise them to review their clinic books which is a paper notebook provided by the health system and commonly kept by patients who see clinicians for long standing conditions in which clinic appointments, clinical assessments, investigations and medications are often detailed. Wellbeing and lifestyle assessment will be collected using the following validated instruments: General Anxiety Disorder scale (GAD-2),[26] Patient Health Questionnaire-2 (PHQ-2),[27] International Physical Activity Questionnaire - Short Form (IPAQ-SF),[28] Audit C for alcohol use,[29] WHO STEPS instrument [30] for assessing tobacco use, Berlin Questionnaire for sleep quality,[31] EQ-5D-5L for quality of life,[32] and the Fried Frailty Index [33]. Diet will also be assessed by asking questions on consumption levels of relevant food groups.[34] Participants will be asked to describe their utilisation of healthcare services in the preceding year, including with public and private services and indigenous healers. Anthropometric measurements will include the recording of each participant's weight, height, waist and hip circumference along with their blood pressure (measured by data collectors). The household survey will take approximately 45 minutes per participant.

Participants will have their heart rhythm recorded using fingertip electrodes which activate a bipolar single lead (lead 1) ECG recording through a smartphone application (AliveCor) that uses an automated algorithm to interpret the cardiac rhythm and identify AF.[35] AliveCor is a validated tool used internationally and is FDA approved.[36,37] Most studies have found the sensitivity and specificity of AliveCor to be 90% or above.[36] The application logs the participants anonymously, assigning a unique ID to each participant and stores a report of the rhythm detected. The participant's unique ID is entered into the data collection tool to enable linkage of the participant's heart rhythm results and their questionnaire data. Reports of the single lead recording will be downloaded and reviewed by a consultant cardiologist off-site. Any participant with a positive or undifferentiated AliveCore results will be referred to see

For peer review only

the study consultant cardiologist and have a 12-lead ECG and relevant assessment. A member of the research team will follow-up with participants that had not visited the hospital within 3 months following referral. Patients access services through the existing government health service.

Digital data collection tools that had been developed and successfully deployed in Sri Lanka were adapted for use in this study.[38] We improved data quality and standardised terminology for diagnosis and comorbidities and included automatic data-logic checks for numerical values to reduce data entry errors. The final tool, utilising the REDCap (Research Electronic Data Capture) [39] software, includes satellite mapping and inbuilt date and time stamps, and supports study coordinators with monitoring data collection and feedback. Data collected is automatically transferred to analytical dashboards (figure 1); thus, the study research team has in near-real time access to study recruitment, data quality, reasons for failure to consent and feasibility of study components. The digital tool is accessible via a mobile device and includes offline functionality, enabling the ability to overcome any issues with mobile data coverage.

The questionnaire and study tools were designed in English and then translated to Tamil by two independent translators. These translations were then reviewed by a working group of bilingual stakeholders from a range of academic, education and professional backgrounds to ensure that the translation and phrasing of the questionnaire was appropriate for monolingual participants. The refined Tamil version of the questionnaire was then back translated to English and reviewed for integrity and retention of information by the study team.

For every cluster GN, a community volunteer will facilitate the data collection by introducing the data collectors to participants. Each data collection team will consist of a trained data collector and the community volunteer. Data collectors will be trained by researchers and community physicians from the University of Jaffna in study participation, consent process, administering the questionnaire and data entry using the digital data collection tool. Data collectors will also be trained in how to administer AliveCor for detecting AF. Trained data collectors will provide information to the participant regarding the study, and obtain written informed consent.

Statistical methods

A direct standardisation technique will be used to obtain province-level prevalence data from

For peer review only

the district level results. The proportion of population exposed to known risk factors of AF will be calculated. Prevalence of risk factors in participants with established, without and with newly diagnosed AF will be determined. Multivariate logistic regressions will be used to calculate adjusted odd ratios for AF risk factors. Population attributable fractions will be calculated for risk factors that were included in the logistic regression model. Statistical analysis would be carried out using SPSS Version 23 (IBM Corp. 2015; Armonk, NY). Raw data will be reported along with scores from assessment tools (e.g GAD-2 and PHQ-2), derived using published definitions. All calculations will be published with open access, ensuring reproducibility of study findings.

Study monitoring

Data quality (completeness, accuracy, logic) will be reviewed daily by the study's data quality coordinators from a central monitoring platform. The study will adhere to the FAIR guiding principles of data management and sharing.[40] In-built dashboards will provide accrued metadata on screening for AF, eligibility, participant recruitment, data quality and prevalence of AF and other noncommunicable diseases in the study population.[38,41] Weekly debriefs between the study team and data collectors will be used to discuss progress and address any challenges arising. In addition, source data will be verified in 1% of questionnaires at random each month where research fellows will join the data collectors and observe study conduct and identify any potential biases in data collection which may impact interrater reliability.

Data storage and security

Data will be stored on institutional network drives of the Information and Communication Technology Agency Sri Lanka, adhering to national data storage requirements.[42] Access to records and data will be limited to study personnel. All participant data will be anonymised by removing any identifiable data from the data collected in field.[43] The data may be linked using a serial number.

Patient and public involvement

Community engagement workshops with key stakeholders were conducted by the study team. Medical Officers of Health (MOHs) and Public Health Midwives (PHMs) within each GN division were asked to participate and aid in the community engagement activities. Stakeholders included patient representatives and primary health care physicians, community health workers and specialist physicians working in the Northern Province. Stakeholders

For peer review only

were asked to give their opinions on the research objectives and their perceptions of potential challenges of conducting large scale epidemiological studies in the province. This included how to achieve participant engagement from marginalised communities, improve health literacy and health seeking behaviours and how to optimise data quality and impact population health policy.[44] These insights were used to inform the selection of study tools for exploring health seeking behaviours, lifestyle assessment and participant information. Similar workshops are planned at three-month intervals during participant enrollment, data collection and analysis, whereby emerging enablers and barriers to the study will be disseminated to stakeholders. Thus, we will create a dialog whereby perceptions of current services can be evaluated and priorities for improvement be identified and agreed with stakeholders throughout the study. Participants from the stakeholder group will be asked to join a smaller working group who will guide the development of health education materials pertaining to lifestyle, general wellbeing and AF specific management. They will provide feedback on the materials, their acceptability, potential barriers to the adoption of new knowledge by the Northern Province community and codesign future implementation methods of the materials.

COVID-19 pandemic

Whilst the pandemic was emerging in Europe and the US, COVID-19 cases were not yet present in Sri Lanka. We utilised this opportunity to discuss perceptions of the disease at the community engagement workshops, assess risk and adapt data collection procedures to reduce risk of transmission. Preventive measures such as hand washing techniques, maintaining social distancing and wearing face masks were explained to data collectors. Hand sanitiser was distributed to all data collectors and they were instructed to disinfect their hands frequently.

ETHICS AND DISSEMINATION

The study received approval from the Ethics Review Committee of Faculty of Medicine, University of Jaffna (J/ERC/19/103/NDR/0208). Written informed consent will be obtained from all participants. A helpline for participants and family members and caretakers of the participants will be set up for any questions or concerns throughout and beyond the study period.

For peer review only

Any participant that is newly diagnosed with AF through this study will be referred to see the study consultant cardiologist. All participants and their families will be invited to use a purpose developed helpline, specifically established for this cohort. The helpline provides advice for participants and their families regarding health, wellbeing, ongoing comorbidity in addition to management of AF. Additionally, research fellows are trained to provide information and advice on lifestyle behaviours where appropriate, and where the participant should go for medical help for any health-related issues.

Results of the study will be made available to participants and/or their caregivers, as appropriate. The findings will be disseminated through abstract presentations at national and international conferences and a manuscript publication in a peer-reviewed journal.

DISCUSSION

This study will be the first of its kind in Sri Lanka and one of few community-based AF screening studies conducted in South Asia.[2] Our findings will determine the prevalence of AF within the Sri Lankan community and enable early and effective treatment for those newly diagnosed with AF. Hospital data in Sri Lanka and other LMICs are often limited, is of poor-quality and typically does not represent the true burden of conditions due to various reasons (healthcare seeking behaviours, lack of diagnostic equipment in primary care, inefficient referral systems, patients lost to follow-up etc.).[45-47] This study will also highlight key enablers and barriers of implementing a population-based screening programme for AF in an LMIC setting, facilitating other LMICs with the means to adapt and implement similar programmes. Dissemination of these findings will provide evidence for policy makers and healthcare professionals of the true burden of AF within the communities they serve, enabling a discourse on how to develop effective care and management to reduce incident AF and improve timely diagnosis of existing AF.

Further to the study aims, recruitment of the 10,000 participants will form a distinct cohort of community members that can be utilised in future studies. All participants have been asked for consent to conducting further analysis with the data collected and to being contacted for future trials and surveys regarding AF and other noncommunicable diseases. To our knowledge, no AF community-based cohort currently exists in Southeast Asia. This study along with future studies carried out within this cohort will enhance the epidemiological evidence on noncommunicable diseases in Sri Lanka. Subsequently, interventions can be

For peer review only

developed and tested for improving the health and wellbeing of LMIC occupants, including those most vulnerable and disadvantaged.

The strengths of this study include the use of mHealth technology for screening for and diagnosing AF in an LMIC setting. In high-income countries the tool has been shown to be easy to use, cost-effective and yields a high accuracy rate.[36] Success of using this tool within the study will indicate the feasibility of using it in other resource-limited settings (including post-war settings) either in the community or in primary care settings that lack appropriate equipment. Conducting this study in an LMIC setting will provide important learnings for future screening programmes in LMICs, extending the current evidence beyond high-income countries. The use of analytical dashboards is another strength of the study. In near-real time, data is able to be reviewed and assessed by the study team both nationally and internationally for any quality issues, enabling immediate rectification and ongoing monitoring. The dashboards also allow for a unique opportunity to review the study findings as data collection progresses. There are however some limitations to be mentioned. One major limitation will be AliveCor's inability to detect paroxysmal AF; however, if feasible and acceptable, the device will offer opportunity to increase rapid detection of AF in community settings of Sri Lanka. Due to the cross-sectional design of this study, only prevalence of AF will be feasible; therefore, AF incidence in the community will remain unknown. Another limitation due to the study design is the inability to infer on the causal relationship between risk factors and AF. Though, the collection of a wide range of demographic and lifestyle data will allow for determination of which risk factors are associated with an existing and new AF diagnosis.

In conclusion, our study will identify the prevalence of AF in the Sri Lankan general population. Further to this, our findings will enable learnings for screening programmes for other LMICs and provide evidence on the feasibility of using a mHealth tool for diagnosing AF in a resource-limited setting. Such learnings have the potential to improve early diagnosis and prevention of AF in LMICs, which will increasingly become a global health priority.

Figure 1. Analytical online dashboard. A breakdown of demographics, lifestyle and wellbeing measures for participants recruited and consented thus far.

For peer review only

Acknowledgements: The authors would like to thank the MOHs, PHMs, data collectors and volunteers for their dedication and invaluable assistance with the pilot study and with recruitment thus far.

Author's contribution: BK, GNT, KN, GYHL, MG, RS, SK, RH, AB and KS conceptualised and designed the study. VAS, PU, CK, SK, BK, RS and KS contributed to the piloting and implementation of the study. SK, TEG and AB drafted the manuscript. All authors reviewed and approved the final manuscript for submission.

Funding: This work was supported by the National Institute for Health Research (project reference 17/63/121, NIHR Global Health Research Group on Atrial Fibrillation Management). The funder had no role in the design of the study or the writing of this protocol.

Competing interests: GYHL is a consultant and speaker for BMS/Pfizer, Boehringer Ingelheim and Daiichi-Sankyo; no fees are received personally. No other authors have any competing interests to declare.

For peer review only

REFERENCES

1. Chugh SS, Roth GA, Gillum RF, et al. Global burden of atrial fibrillation in developed and developing nations. *Glob Heart* 2014;9(1):113-9. doi: 10.1016/j.gheart.2014.01.004 [published Online First: 2014/11/30]
2. Zulkifly H, Lip GY, Lane DA. Epidemiology of atrial fibrillation. *Int J Clin Pract* 2018;72(3):e13070.
3. Björck S, Palaszewski B, Friberg L, et al. Atrial fibrillation, stroke risk, and warfarin therapy revisited: a population-based study. *Stroke* 2013;44(11):3103-08.
4. Katsanos AH, Kamel H, Healey JS, et al. Stroke prevention in atrial fibrillation: looking forward. *Circulation* 2020;142(24):2371-88.
5. Chao T-F, Nedeljkovic MA, Lip GY, et al. Stroke prevention in atrial fibrillation: comparison of recent international guidelines. *Eur Heart J Suppl* 2020;22(Supplement_O):O53-O60.
6. Xiong Q, Proietti M, Senoo K, et al. Asymptomatic versus symptomatic atrial fibrillation: a systematic review of age/gender differences and cardiovascular outcomes. *Int J Cardiol* 2015;191:172-77.
7. Freedman B, Camm J, Calkins H, et al. Screening for atrial fibrillation: a report of the AF-SCREEN International Collaboration. *Circulation* 2017;135(19):1851-67.
8. Hindricks G, Potpara T, Dagres N, et al. 2020 ESC Guidelines for the diagnosis and management of atrial fibrillation developed in collaboration with the European Association for Cardio-Thoracic Surgery (EACTS) The Task Force for the diagnosis and management of atrial fibrillation of the European Society of Cardiology (ESC) Developed with the special contribution of the European Heart Rhythm Association (EHRA) of the ESC. *Eur Heart J* 2021;42(5):373-498.
9. Lee J, Reyes BA, McManus DD, et al. Atrial fibrillation detection using an iPhone 4S. *IEEE Trans Biomed Eng* 2012;60(1):203-06.
10. Orchard J, Lowres N, Freedman SB, et al. Screening for atrial fibrillation during influenza vaccinations by primary care nurses using a smartphone electrocardiograph (iECG): a feasibility study. *Eur J Prev Cardiol* 2016;23(2_suppl):13-20.
11. Bowry AD, Lewey J, Dugani SB, et al. The burden of cardiovascular disease in low-and middle-income countries: epidemiology and management. *Can J Cardiol* 2015;31(9):1151-59.
12. Soni A, Karna S, Patel H, et al. Study protocol for Smartphone Monitoring for Atrial fibrillation in Real-Time in India (SMART-India): a community-based screening and referral programme. *BMJ Open* 2017;7(12):e017668.
13. Davidson KW, Barry MJ, Mangione CM, et al. Screening for atrial fibrillation: US Preventive Services Task Force recommendation statement. *JAMA* 2022;327(4):360-67.
14. Stavrakis S, Elkholey K, Lofgren MM, et al. Screening for atrial fibrillation in American Indian adults in a tribal primary care clinic. *J Am Heart Assoc* 2021;10(9):e020069.
15. Rosengren A, Smyth A, Rangarajan S, et al. Socioeconomic status and risk of cardiovascular disease in 20 low-income, middle-income, and high-income countries: the Prospective Urban Rural Epidemiologic (PURE) study. *Lancet Glob Health* 2019;7(6):e748-e60.
16. Bai Y, Wang Y-L, Shantsila A, et al. The global burden of atrial fibrillation and stroke: a systematic review of the clinical epidemiology of atrial fibrillation in Asia. *Chest* 2017;152(4):810-20.
17. Santos IS, Goulart AC, Olmos RD, et al. Atrial fibrillation in low-and middle-income countries: a narrative review. *Eur Heart J Suppl* 2020;22(Supplement_O):O61-O77.
18. Northern Provincial Council. Vital Statistics 2020. Jaffna, Sri Lanka: Office of the Deputy Chief Secretary, 2020.
19. Central Bank of Sri Lanka. Provincial Gross Domestic Product - 2019. Colombo, Sri Lanka: Statistics Department, 2020.
20. Patino CM, Ferreira JC. Inclusion and exclusion criteria in research studies: definitions and why they matter. *J Bras Pneumol* 2018;44:84-84.

For peer review only - http://bmjopen.bmj.com/site/about/guidelines.xhtml

21. O'Neill J, Tayebjee MH. Why are South Asians seemingly protected against the development of atrial fibrillation? A review of current evidence. *Trends Cardiovasc Med* 2017;27(4):249-57.
22. Chang T, Gajasinghe S, Arambepola C. Prevalence of stroke and its risk factors in urban Sri Lanka: population-based study. *Stroke* 2015;46(10):2965-68.
23. Martínez-Mesa J, González-Chica DA, Bastos JL, et al. Sample size: how many participants do I need in my research? *An Bras Dermatol* 2014;89:609-15.
24. Department of Census and Statistics Sri Lanka. Demographic and Health Survey 2016 Sri Lanka: Department of Census and Statistics Sri Lanka, 2016.
25. Austin SR, Wong Y-N, Uzzo RG, et al. Why summary comorbidity measures such as the Charlson comorbidity index and Elixhauser score work. *Med Care* 2015;53(9):e65.
26. Kroenke K, Spitzer RL, Williams JB, et al. Anxiety disorders in primary care: prevalence, impairment, comorbidity, and detection. *Ann Intern Med* 2007;146(5):317-25.
27. Arroll B, Goodyear-Smith F, Crengle S, et al. Validation of PHQ-2 and PHQ-9 to screen for major depression in the primary care population. *Ann Fam Med* 2010;8(4):348-53.
28. Lee PH, Macfarlane DJ, Lam TH, et al. Validity of the international physical activity questionnaire short form (IPAQ-SF): A systematic review. *Int J Behav Nutr Phys Act* 2011;8(1):1-11.
29. Bush K, Kivlahan DR, McDonell MB, et al. The AUDIT alcohol consumption questions (AUDIT-C): an effective brief screening test for problem drinking. *Arch Intern Med* 1998;158(16):1789-95.
30. World Health Organization. WHO STEPS surveillance manual: the WHO STEPwise approach to chronic disease risk factor surveillance. Geneva: World Health Organization, 2005.
31. Netzer NC, Stoohs RA, Netzer CM, et al. Using the Berlin Questionnaire to identify patients at risk for the sleep apnea syndrome. *Ann Intern Med* 1999;131(7):485-91.
32. Herdman M, Gudex C, Lloyd A, et al. Development and preliminary testing of the new five-level version of EQ-5D (EQ-5D-5L). *Qual Life Res* 2011;20(10):1727-36.
33. Revenig LM, Canter DJ, Kim S, et al. Report of a simplified frailty score predictive of short-term postoperative morbidity and mortality. *J Am Coll Surg* 2015;220(5):904-11. e1.
34. Schooling CM, Ho SY, Leung GM, et al. Diet synergies and mortality—a population-based case–control study of 32 462 Hong Kong Chinese older adults. *Int J Epidemiol* 2006;35(2):418-26.
35. Galloway CD, Albert DE, Freedman SB. iPhone ECG application for community screening to detect silent atrial fibrillation: a novel technology to prevent stroke. *Int J Cardiol* 2013;165:193-94.
36. Hall A, Mitchell ARJ, Wood L, et al. Effectiveness of a single lead AliveCor electrocardiogram application for the screening of atrial fibrillation: a systematic review. *Medicine (Baltimore)* 2020;99(30)
37. Cheung CC, Krahn AD, Andrade JG. The emerging role of wearable technologies in detection of arrhythmia. *Can J Cardiol* 2018;34(8):1083-87.
38. Beane A, De Silva AP, Athapattu PL, et al. Addressing the information deficit in global health: lessons from a digital acute care platform in Sri Lanka. *BMJ Glob Health* 2019;4(1):e001134.
39. REDCap. REDCap: Research Electronic Data Capture Nashville, Tennessee: Vanderbilt University; [Available from: https://projectredcap.org/.
40. Wilkinson MD, Dumontier M, Aalbersberg IJ, et al. The FAIR Guiding Principles for scientific data management and stewardship. *Sci Data* 2016;3(1):1-9.
41. CRIT Care Asia, Hashmi M, Beane A, et al. Leveraging a cloud-based critical care registry for COVID-19 pandemic surveillance and research in low-and middle-income countries. *JMIR Public Health Surveill* 2020;6(4):e21939.
42. Information and Communication Technology Agency of Sri Lanka. Security Sri Lanka: Information and Communication Technology Agency of Sri Lanka; [Available from: https://www.icta.lk/security/.

For peer review only

43. Centers for Disease Control Prevention. HIPAA privacy rule and public health. Guidance from CDC and the US Department of Health and Human Services. *MMWR Morb Mortal Wkly Rep* 2003;52(Suppl 1):1-17, 19.
44. Cook N, Siddiqi N, Twiddy M, et al. Patient and public involvement in health research in low and middle-income countries: a systematic review. *BMJ Open* 2019;9(5):e026514.
45. Arsenault C, Kim MK, Aryal A, et al. Hospital-provision of essential primary care in 56 countries: determinants and quality. *Bull World Health Organ* 2020;98(11):735.
46. Chow CK, Nguyen TN, Marschner S, et al. Availability and affordability of medicines and cardiovascular outcomes in 21 high-income, middle-income and low-income countries. *BMJ Glob Health* 2020;5(11):e002640.
47. Lowres N, Giskes K, Hespe C, et al. Reducing stroke risk in atrial fibrillation: adherence to guidelines has improved, but patient persistence with anticoagulant therapy remains suboptimal. *Korean Circ J* 2019;49(10):883-907.

For peer review only

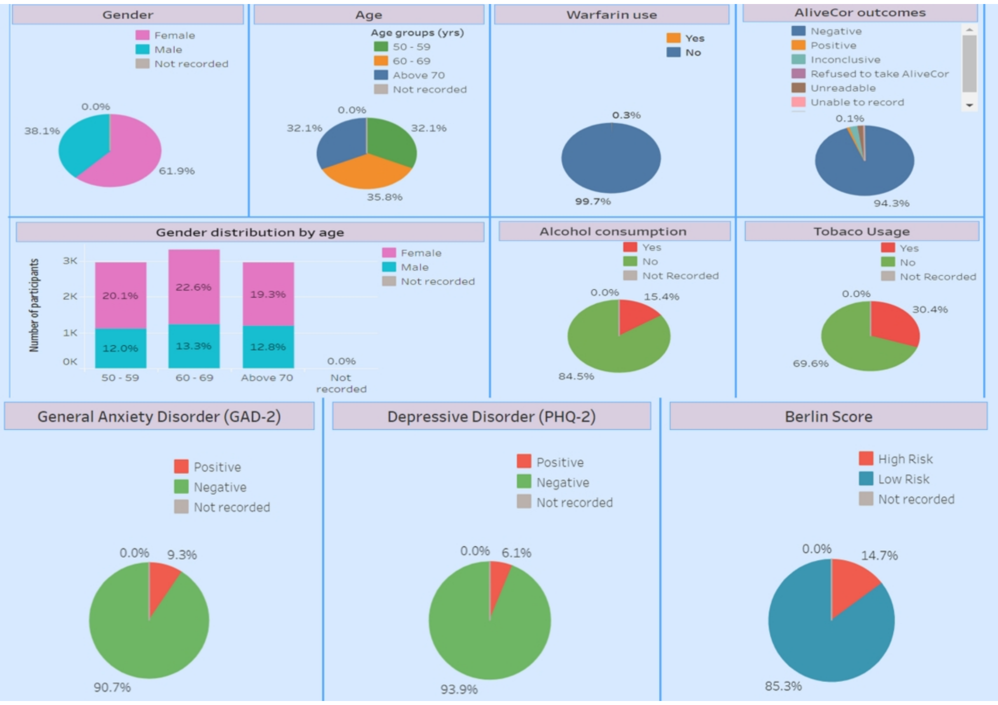

Analytical online dashboard. A breakdown of demographics, lifestyle and wellbeing measures for participants recruited and consented thus far.

BMJ Open

Prevalence of atrial fibrillation in Northern Sri Lanka: A study protocol for a cross-sectional household survey

Journal:	*BMJ Open*
Manuscript ID	bmjopen-2021-056480.R2
Article Type:	Protocol
Date Submitted by the Author:	30-Sep-2022
Complete List of Authors:	Kanesamoorthy, Shribavan; University of Jaffna Faculty of Medicine, Department of Community & Family Medicine<br>Sheron, Vethanayagam Antony; University of Jaffna Faculty of Medicine, Department of Community & Family Medicine<br>Uruthirakumar, Powsiga; University of Jaffna Faculty of Medicine, Department of Community & Family Medicine<br>Kodippily, Chamira; National Intensive Care Surveillance-Mahidol Oxford Tropical Medicine Research Unit<br>Kumarendran, Balachandran; University of Jaffna Faculty of Medicine, Department of Community & Family Medicine<br>Gooden, Tiffany; University of Birmingham College of Medical and Dental Sciences, Department of Public Health and Epidemiology<br>Thomas, G. Neil; University of Birmingham College of Medical and Dental Sciences, Department of Public Health and Epidemiology<br>Nirantharakumar, Krishnarajah; University of Birmingham College of Medical and Dental Sciences, Department of Public Health and Epidemiology<br>Lip, Gregory; University of Birmingham College of Medical and Dental Sciences, Department of Public Health and Epidemiology; University of Liverpool, Liverpool Centre for Cardiovascular Science<br>Guruparan, Mahesan; Jaffna Teaching Hospital<br>Haniffa, Rashan; The University of Edinburgh Centre for Inflammation Research<br>Surenthirakumaran, Rajendra; University of Jaffna Faculty of Medicine, Department of Community & Family Medicine<br>Beane, Abi; The University of Edinburgh Centre for Inflammation Research<br>Subaschandran, Kumaran; University of Jaffna Faculty of Medicine, Department of Community & Family Medicine
**Primary Subject Heading**:	Diagnostics
Secondary Subject Heading:	Cardiovascular medicine, Epidemiology, Global health, Research methods
Keywords:	Cardiac Epidemiology < CARDIOLOGY, Telemedicine < BIOTECHNOLOGY & BIOINFORMATICS, Adult cardiology < CARDIOLOGY, Cardiology < INTERNAL MEDICINE, STATISTICS & RESEARCH METHODS, PREVENTIVE MEDICINE

For peer review only - http://bmjopen.bmj.com/site/about/guidelines.xhtml

For peer review only - http://bmjopen.bmj.com/site/about/guidelines.xhtml

Prevalence of atrial fibrillation in Northern Sri Lanka: A study protocol for a cross-sectional household survey

Shribavan Kanesamoorthy[1], Vethanayagam Antony Sheron[1], Powsiga Uruthirakumar[1], Chamira Kodippily[2], Balachandran Kumarendran[1], Tiffany E Gooden[3], G Neil Thomas[3*], Krishnarajah Nirantharakumar[3], Gregory YH Lip[3,4*], Mahesan Guruparan[5], Rashan Haniffa[2], Rajendra Surenthirakumaran[1*□], Abi Beane[2*], Kumaran Subaschandran[1*□] on behalf of the NIHR Global Health Research Group on Atrial Fibrillation Management^

1. Department of Community & Family Medicine, Faculty of Medicine, University of Jaffna, Kokuvil, Sri Lanka

2. National Intensive Care Surveillance-Mahidol Oxford Tropical Medicine Research Unit, Jaffna, Sri Lanka

3. Institute of Applied Health Research, University of Birmingham, Birmingham, United Kingdom

4. Liverpool Centre for Cardiovascular Science, University of Liverpool, Liverpool John Moores University and Liverpool Heart & Chest Hospital, Liverpool, United Kingdom; and Department of Clinical Medicine, Aalborg University, Aalborg, Denmark

5. Handy Memorial Cardiology Unit, Jaffna Teaching Hospital, Jaffna, Sri Lanka

* Profs Lip and Thomas are co-Directors of the NIHR Global Health Research Group on Atrial Fibrillation Management, and are joint senior authors with Drs Surenthirakumaran, Beane and Subaschandraen.

□ Joint corresponding authors:
G Neil Thomas: g.n.thomas@bham.ac.uk/gneilthomas@gmail.com University of Birmingham, Edgbaston, Birmingham, United Kingdom

Rajendra Surenthirakumaran: surenthirakumaran@gmail.com University of Jaffna, Aadiyapatham Road, Kokuvil, Jaffna, Sri Lanka

^ Members of the NIHR Global Health Group on Atrial Fibrillation Management listed in alphabetical order: Ajini Arasalingam, Abi Beane, Isabela M Bensenor, Peter Brocklehurst, Kar Keung Cheng, Wahbi El-Bouri, Mei Feng, Alessandra C Goulart, Sheila Greenfield, Yutao Guo, Mahesan Guruparan, Gustavo Gusso, Tiffany E Gooden, Rashan Haniffa, Lindsey Humphreys, Kate Jolly, Sue Jowett, Chamira Kodippily, Balachandran Kumarendran, Emma Lancashire, Deirdre A Lane, Xuewen Li, Gregory Y.H. Lip (Co-PI), Yan-guang Li, Trudie Lobban, Paulo A Lotufo, Semira Manaseki-Holland, David Moore,

For peer review only

Krishnarajah Nirantharakumar, Rodrigo D Olmos, Elisabete Paschoal, Paskaran Pirasanth, Uruthirakumar Powsiga, Carla Romagnolli, Itamar S Santos, Alena Shantsila, Vethanayagan Antony Sheron, Kanesamoorthy Shribavan, Isabelle Szmigin, Kumaran Subaschandren, Rajendra Surenthirakumaran, Meihui Tai, G. Neil Thomas (Co-PI), Timo Tolppa, Ana C Varella, Hao Wang, Jingya Wang, Hui Zhang, Jiaoyue Zhong.

Word count: 3494

Keywords: atrial fibrillation, low- and middle-income countries, epidemiological studies, community-based survey, screening programme, noncommunicable diseases

For peer review only

ABSTRACT

Introduction: Atrial fibrillation (AF) is the most common arrhythmia globally. It is associated with a 5-fold risk in stroke, but early diagnosis and effective treatment can reduce this risk. AF is often underdiagnosed, particularly in low- and middle-income countries (LMICs) where screening for AF is not always feasible or considered common practice in primary care settings. Epidemiological data on AF in LMICs is often incomplete particularly in vulnerable populations. This LMIC research collaborative aims to identify the prevalence of AF in the Northern Sri Lankan community.

Methods and analysis: A cross-sectional household survey piloted and codesigned through a series of community engagement events will be administered in all five districts in Northern Province, Sri Lanka. A multistage cluster sampling approach will be used starting at district level, then the Divisional Secretariats followed by Grama Niladhari divisions. Twenty households will be selected from each cluster. The study aims to recruit 10,000 participants aged 50 years or older, one participant per household. Demographic and socioeconomic characteristics, wellbeing and lifestyle and anthropometric measurements will be collected using a digital data platform (REDCap) by trained data collectors. Participants will be screened for AF using a fingertip single lead ECG via a smartphone application (AliveCor) with rhythm strips reviewed by a consultant cardiologist. Prevalence of AF and risk factors will be established at province and district-levels. Adjusted odds ratios and population attributable fractions for AF risk factors will be determined.

Ethics and dissemination: This study was approved by the Ethics Review Committee of Faculty of Medicine at University of Jaffna. Written informed consent will be obtained from all participants. Findings will be disseminated through publication in a peer-reviewed journal and presentations at conferences. The findings will enable early treatment for new AF diagnoses and inform interventions to improve community-based management of AF in LMICs.

For peer review only

STRENGTHS AND LIMITATIONS OF THIS STUDY

- This study utilises mHealth technology that has the ability to quickly and efficiently identify atrial fibrillation (AF) within resource-limited settings.
- The use of a near-real time data collection tool and analytical dashboard will enable the ability to identify and rectify any data collection issues as the study progresses.
- Conducting the study in this LMIC setting will inform future noncommunicable screening policies to extend the evidence base beyond high-income settings.
- Whilst data pertaining to a variety of risk factors will be collected, the temporal relationship between risk factors and AF will not be attained.
- Given the cross-sectional study design, incidence of AF and other noncommunicable diseases will not be feasible.

peer review only

INTRODUCTION

Atrial fibrillation (AF) is the commonest cardiac rhythm disorder globally.[1] AF prevalence is increasing worldwide with age, male sex, hypertension, diabetes and heart disease as major risk factors.[2] AF is associated with a 5-fold increase in stroke and over 25% of strokes are due to AF.[3] Early identification and effective management of AF can prevent strokes and contribute to improved management of cardiovascular health.[3-5] Stroke caused by AF is largely preventable with appropriate use of oral anticoagulant (OAC) therapy, a therapy which is readily available in health systems internationally.[5] However, an estimated 33% of people with AF are asymptomatic, meaning many people with AF remain undiagnosed until they present with life threatening complications.[6]

Screening for AF in people 65 years or older is now recommended by some clinical practice guidelines and is common place in general practice in upper-middle and high-income countries.[7,8] Such population screening has in part been made possible by the successful development and scale up of smartphone technologies, which enable rapid point of care diagnosis through opportunistic pulse palpation or ECG rhythm strip.[9,10]

Such practice remains limited in low- and middle-income countries (LMICs), where cardiovascular diseases are being increasingly reported in younger populations (ages 50 years and up), and which are rapidly attributing to causes of mortality.[11-14] Chronic disease prevalence in this relatively young population appears as a result of both childhood conditions such as rheumatic fever and valvular heart disease, and early onset of diabetes and hypertension as a result of age and lifestyle-related factors.[11,15] Additionally, complications associated with noncommunicable diseases are attributed with worse outcomes and greater morbidity for people living in LMICs when compared to high-income settings.[16] Despite this disparity in diagnosis, management and outcomes, there is limited high quality epidemiological data on AF in LMICs, specifically in South Asia.[2,15]

Screening programmes in the regions are hindered by poor recruitment, often as a result of poor community engagement and low levels of noncommunicable disease-related health literacy. Where programmes do exist, vulnerable groups are often underrepresented and missing or incomplete data inhibits meaningful interpretation of data.[17] Epidemiology researchers are increasingly looking to social science and implementation strategies to engage communities in research design to ensure they are included in such programmes. Particularly

For peer review only - http://bmjopen.bmj.com/site/about/guidelines.xhtml

in LMICs, it is imperative to overcome these known barriers to study implementation and ensure that study findings are not only disseminated back to communities, but are both acceptable to and utilised by healthcare providers.

This study aims to implement an AF screening programme using smartphone technology, feasible for delivery at scale in resource constrained LMIC settings. Specifically, we aim to determine the prevalence of AF and associated risk factors in Northern Province, Sri Lanka and to evaluate the feasibility and acceptability of smartphone enabled single lead electrocardiogram (ECG) for the purpose of diagnosing AF.

METHODS AND ANALYSIS

Research design

This cross-sectional study combines a household questionnaire and an onsite assessment of the presence of AF using a single lead ECG reader on a digital mobile health (mHealth) platform. The study commenced in June 2020 and is expected to be complete by March 2022.

Study setting

This study is being conducted across all five districts in the Northern Province of Sri Lanka: Jaffna, Kilinochchi, Mannar, Mullaitivu and Vavuniya. Each district is divided into administrative units called Divisional Secretariats (DS). Each DS is further divided into many Grama Niladhari (GN) divisions. Each GN division is identified by a unique number. Northern Province is located just 22 miles (35 km) southeast of India and has approximately 1.3 million permanent residents.[18] Its climate is typically tropical, but with a hot dry season from February to September. Whilst the Northern Province was heavily impacted by 30 years of conflict, the province has recently opened to the global market and its share of Provincial Gross Domestic Product is increasing.[19] Publicly available healthcare including medical appointments, medications and medical procedures are free to all Sri Lankan citizens.

Sampling and sample size

Consistent with other LMIC studies and in response to the increasing literature on morbidity and chronic disease prevalence in South Asia, we selected a threshold of 50 years and above for inclusion in screening, a lower than current recommended practice of 65 years.[12] Therefore, individuals aged 50 years or above will be considered eligible; additionally, they

For peer review only

must be fluent in the Tamil language. Based on already published methods,[20] anyone suffering from terminal illnesses, in need of immediate hospital admission or currently an inpatient in a hospital setting will be excluded. All data collectors – which includes a medically trained doctor and two nursing graduates – will be trained to assess individuals for eligibility.

This study will use a multistage cluster sampling approach. The multistage process will start at the district level, then to the DS level followed by the GN level. GN divisions will then be divided into clusters based on population size and from these clusters, a single cluster will be selected at random. Each cluster will consist of 20 households, one participant per household. We will first randomly select an index house within each cluster and from the index house, 20 households on the right side will be selected. In households where more than one eligible person lives, the person with a birthday closest to the date of visit will be selected. We used 2012 population census data to select the clusters and index house within each cluster.[19]

For the sample size calculation, the prevalence of AF in Sri Lanka was estimated to be 1% based on previous evidence.[2,21,22] We used a design effect of 2 to adjust for cluster sampling, and we used an alpha level of 5% and beta level of 20%. We increased our sample size by 10% to account for non-participation,[23] leading to a minimum sample size of 10,000 participants required.

Pilot study

The data collection tool was piloted in 100 households located in a GN division that will not be included within the main study. The pilot study comprised participants from varying socioeconomic backgrounds and education levels. Participants were asked to give their perspectives on acceptability of participating in the study including the duration of the interview, ease of understanding the interviewer administered questionnaire and their experience of using the fingertip rhythm assessment ECG application. Additionally, data collectors fed back on the challenges they experienced in collecting the data and reported on their perception of participants' willingness to participate and recall, internal consistency reliability and interrater reliability of the questionnaire. During the piloting of the questionnaire, participants' questions and concerns were noted and reported through the electronic data collection tool used; these were reviewed daily by the principal and co-investigators. Feedback following the pilot revealed i, data collector concerns regarding availability of and access to clinic books for relevant medical history, ii, participant concerns

For peer review only

regarding future use of fingerprints, reasons for the survey and being disadvantaged if they were found to have AF, and iii, participants understanding of medical prescriptions. In response, we made the following adaptations to the study conduct protocol prior to commencing data collection: reporting of medical history will be reported as either being directly observed from clinic books or via participant recall; medications will be reported by categories for simplicity and; an update of participant information leaflets to insure the aim of the study, data security and what happens after the study is clear. Additionally, further training for the data collection team was provided on the management of personal information, the AliveCor device and how to respond to participant concerns.

Data collection

The household survey comprises five sections: i) demographics and socioeconomic profile; ii) current and past health conditions; iii) wellbeing and lifestyle; iv) anthropometric measurements and; v) screening for AF. The demographic and socioeconomic profile will be collected using the Sri Lanka Demographic and Health Survey 2016 questionnaire which includes education levels and occupation.[24] Current medical conditions and medical history of comorbid diseases will be collected using the Charlson Comorbidity Index [25] along with the recording of any current medications prescribed by physicians or indigenous healers. To assist participant's recall of their medical history, the data collection team will advise them to review their clinic books which is a paper notebook provided by the health system and commonly kept by patients who see clinicians for long standing conditions in which clinic appointments, clinical assessments, investigations and medications are often detailed. Wellbeing and lifestyle assessment will be collected using the following validated instruments: General Anxiety Disorder scale (GAD-2),[26] Patient Health Questionnaire-2 (PHQ-2),[27] International Physical Activity Questionnaire - Short Form (IPAQ-SF),[28] Audit C for alcohol use,[29] WHO STEPS instrument [30] for assessing tobacco use, Berlin Questionnaire for sleep quality,[31] EQ-5D-5L for quality of life,[32] and the Fried Frailty Index [33]. Diet will also be assessed by asking questions on consumption levels of relevant food groups.[34] Participants will be asked to describe their utilisation of healthcare services in the preceding year, including with public and private services and indigenous healers. Anthropometric measurements will include the recording of each participant's weight, height, waist and hip circumference along with their blood pressure (measured by data collectors). The household survey will take approximately 45 minutes per participant.

For peer review only

Participants will have their heart rhythm recorded using fingertip electrodes which activate a bipolar single lead (lead 1) ECG recording through a smartphone application (AliveCor) that uses an automated algorithm to interpret the cardiac rhythm and identify AF.[35] AliveCor is a validated tool used internationally and is FDA approved.[36,37] Most studies have found the sensitivity and specificity of AliveCor to be 90% or above.[36] The application logs the participants anonymously, assigning a unique ID to each participant and stores a report of the rhythm detected. The participant's unique ID is entered into the data collection tool to enable linkage of the participant's heart rhythm results and their questionnaire data. Reports of the single lead recording (AliveCor report) will be downloaded and reviewed by a consultant cardiologist off-site who will determine the outcome of the test. Any participant with a positive or undifferentiated AliveCor results will be referred to see the study consultant cardiologist and have a 12-lead ECG and relevant assessment. A member of the research team will follow-up with participants that had not visited the hospital within 3 months following referral. Patients access services through the existing government health service.

Digital data collection tools that had been developed and successfully deployed in Sri Lanka were adapted for use in this study.[38] We improved data quality and standardised terminology for diagnosis and comorbidities and included automatic data-logic checks for numerical values to reduce data entry errors. The final tool, utilising the REDCap (Research Electronic Data Capture) [39] software, includes satellite mapping and inbuilt date and time stamps, and supports study coordinators with monitoring data collection and feedback. Data collected is automatically transferred to analytical dashboards (figure 1); thus, the study research team has in near-real time access to study recruitment, data quality, reasons for failure to consent and feasibility of study components. The digital tool is accessible via a mobile device and includes offline functionality, enabling the ability to overcome any issues with mobile data coverage.

The questionnaire and study tools were designed in English and then translated to Tamil by two independent translators. These translations were then reviewed by a working group of bilingual stakeholders from a range of academic, education and professional backgrounds to ensure that the translation and phrasing of the questionnaire was appropriate for monolingual participants. The refined Tamil version of the questionnaire was then back translated to English and reviewed for integrity and retention of information by the study team.

For peer review only

For every cluster GN, a community volunteer will facilitate the data collection by introducing the data collectors to participants. Each data collection team will consist of a trained data collector and the community volunteer. Data collectors will be trained by researchers and community physicians from the University of Jaffna in study participation, consent process, administering the questionnaire and data entry using the digital data collection tool. Data collectors will also be trained in how to administer AliveCor for detecting AF. Trained data collectors will provide information to the participant regarding the study, and obtain written informed consent.

Statistical methods

A direct standardisation technique will be used to obtain province-level prevalence data from the district level results. The proportion of population exposed to known risk factors of AF will be calculated. Prevalence of risk factors in participants with established, without and with newly diagnosed AF will be determined. Multivariate logistic regressions will be used to calculate adjusted odd ratios for AF risk factors. Population attributable fractions will be calculated for risk factors that were included in the logistic regression model. Statistical analysis would be carried out using SPSS Version 23 (IBM Corp. 2015; Armonk, NY). Raw data will be reported along with scores from assessment tools (e.g GAD-2 and PHQ-2), derived using published definitions. All calculations will be published with open access, ensuring reproducibility of study findings.

Study monitoring

Data quality (completeness, accuracy, logic) will be reviewed daily by the study's data quality coordinators from a central monitoring platform. The study will adhere to the FAIR guiding principles of data management and sharing.[40] In-built dashboards will provide accrued metadata on screening for AF, eligibility, participant recruitment, data quality and prevalence of AF and other noncommunicable diseases in the study population.[38,41] Weekly debriefs between the study team and data collectors will be used to discuss progress and address any challenges arising. In addition, source data will be verified in 1% of questionnaires at random each month where research fellows will join the data collectors and observe study conduct and identify any potential biases in data collection which may impact interrater reliability.

Data storage and security

Data will be stored on institutional network drives of the Information and Communication

For peer review only

Technology Agency Sri Lanka, adhering to national data storage requirements.[42] Access to records and data will be limited to study personnel. All participant data will be anonymised by removing any identifiable data from the data collected in field.[43] The data may be linked using a serial number.

Patient and public involvement

Community engagement workshops with key stakeholders were conducted by the study team. Medical Officers of Health (MOHs) and Public Health Midwives (PHMs) within each GN division were asked to participate and aid in the community engagement activities. Stakeholders included patient representatives and primary health care physicians, community health workers and specialist physicians working in the Northern Province. Stakeholders were asked to give their opinions on the research objectives and their perceptions of potential challenges of conducting large scale epidemiological studies in the province. This included how to achieve participant engagement from marginalised communities, improve health literacy and health seeking behaviours and how to optimise data quality and impact population health policy.[44] These insights were used to inform the selection of study tools for exploring health seeking behaviours, lifestyle assessment and participant information. Similar workshops are planned at three-month intervals during participant enrollment, data collection and analysis, whereby emerging enablers and barriers to the study will be disseminated to stakeholders. Thus, we will create a dialog whereby perceptions of current services can be evaluated and priorities for improvement be identified and agreed with stakeholders throughout the study. Participants from the stakeholder group will be asked to join a smaller working group who will guide the development of health education materials pertaining to lifestyle, general wellbeing and AF specific management. They will provide feedback on the materials, their acceptability, potential barriers to the adoption of new knowledge by the Northern Province community and codesign future implementation methods of the materials.

COVID-19 pandemic

Whilst the pandemic was emerging in Europe and the US, COVID-19 cases were not yet present in Sri Lanka. We utilised this opportunity to discuss perceptions of the disease at the community engagement workshops, assess risk and adapt data collection procedures to reduce risk of transmission. Preventive measures such as hand washing techniques, maintaining social distancing and wearing face masks were explained to data collectors.

For peer review only

Hand sanitiser was distributed to all data collectors and they were instructed to disinfect their hands frequently.

ETHICS AND DISSEMINATION

The study received approval from the Ethics Review Committee of Faculty of Medicine, University of Jaffna (J/ERC/19/103/NDR/0208). Written informed consent will be obtained from all participants. A helpline for participants and family members and caretakers of the participants will be set up for any questions or concerns throughout and beyond the study period.

Any participant that is newly diagnosed with AF through this study will be referred to see the study consultant cardiologist. All participants and their families will be invited to use a purpose developed helpline, specifically established for this cohort. The helpline provides advice for participants and their families regarding health, wellbeing, ongoing comorbidity in addition to management of AF. Additionally, research fellows are trained to provide information and advice on lifestyle behaviours where appropriate, and where the participant should go for medical help for any health-related issues.

Results of the study will be made available to participants and/or their caregivers, as appropriate. The findings will be disseminated through abstract presentations at national and international conferences and a manuscript publication in a peer-reviewed journal.

DISCUSSION

This study will be the first of its kind in Sri Lanka and one of few community-based AF screening studies conducted in South Asia.[2] Our findings will determine the prevalence of AF within the Sri Lankan community and enable early and effective treatment for those newly diagnosed with AF. Hospital data in Sri Lanka and other LMICs are often limited, is of poor-quality and typically does not represent the true burden of conditions due to various reasons (healthcare seeking behaviours, lack of diagnostic equipment in primary care, inefficient referral systems, patients lost to follow-up etc.).[45-47] This study will also highlight key enablers and barriers of implementing a population-based screening programme for AF in an LMIC setting, facilitating other LMICs with the means to adapt and implement similar programmes. Dissemination of these findings will provide evidence for policy makers and healthcare professionals of the true burden of AF within the communities they serve,

For peer review only

enabling a discourse on how to develop effective care and management to reduce incident AF and improve timely diagnosis of existing AF.

Further to the study aims, recruitment of the 10,000 participants will form a distinct cohort of community members that can be utilised in future studies. All participants have been asked for consent to conducting further analysis with the data collected and to being contacted for future trials and surveys regarding AF and other noncommunicable diseases. To our knowledge, no AF community-based cohort currently exists in Southeast Asia. This study along with future studies carried out within this cohort will enhance the epidemiological evidence on noncommunicable diseases in Sri Lanka. Subsequently, interventions can be developed and tested for improving the health and wellbeing of LMIC occupants, including those most vulnerable and disadvantaged.

The strengths of this study include the use of mHealth technology for screening for and diagnosing AF in an LMIC setting. In high-income countries the tool has been shown to be easy to use, cost-effective and yields a high accuracy rate.[36] Success of using this tool within the study will indicate the feasibility of using it in other resource-limited settings (including post-war settings) either in the community or in primary care settings that lack appropriate equipment. Conducting this study in an LMIC setting will provide important learnings for future screening programmes in LMICs, extending the current evidence beyond high-income countries. The use of analytical dashboards is another strength of the study. In near-real time, data is able to be reviewed and assessed by the study team both nationally and internationally for any quality issues, enabling immediate rectification and ongoing monitoring. The dashboards also allow for a unique opportunity to review the study findings as data collection progresses. There are however some limitations to be mentioned. One major limitation will be AliveCor's inability to detect paroxysmal AF; however, if feasible and acceptable, the device will offer opportunity to increase rapid detection of AF in community settings of Sri Lanka. Due to the cross-sectional design of this study, only prevalence of AF will be feasible; therefore, AF incidence in the community will remain unknown. Another limitation due to the study design is the inability to infer on the causal relationship between risk factors and AF. Though, the collection of a wide range of demographic and lifestyle data will allow for determination of which risk factors are associated with an existing and new AF diagnosis.

For peer review only

In conclusion, our study will identify the prevalence of AF in the Sri Lankan general population. Further to this, our findings will enable learnings for screening programmes for other LMICs and provide evidence on the feasibility of using a mHealth tool for diagnosing AF in a resource-limited setting. Such learnings have the potential to improve early diagnosis and prevention of AF in LMICs, which will increasingly become a global health priority.

Figure 1. Analytical online dashboard. A breakdown of demographics, lifestyle and wellbeing measures for participants recruited and consented thus far.

Acknowledgements: The authors would like to thank the MOHs, PHMs, data collectors and volunteers for their dedication and invaluable assistance with the pilot study and with recruitment thus far.

Author's contribution: BK, GNT, KN, GYHL, MG, RS, SK, RH, AB and KS conceptualised and designed the study. VAS, PU, CK, SK, BK, RS and KS contributed to the piloting and implementation of the study. SK, TEG and AB drafted the manuscript. All authors reviewed and approved the final manuscript for submission.

Funding: This work was supported by the National Institute for Health Research (project reference 17/63/121, NIHR Global Health Research Group on Atrial Fibrillation Management). The funder had no role in the design of the study or the writing of this protocol.

Competing interests: GYHL is a consultant and speaker for BMS/Pfizer, Boehringer Ingelheim and Daiichi-Sankyo; no fees are received personally. No other authors have any competing interests to declare.

For peer review only

REFERENCES

1. Chugh SS, Roth GA, Gillum RF, et al. Global burden of atrial fibrillation in developed and developing nations. *Glob Heart* 2014;9(1):113-9. doi: 10.1016/j.gheart.2014.01.004 [published Online First: 2014/11/30]
2. Zulkifly H, Lip GY, Lane DA. Epidemiology of atrial fibrillation. *Int J Clin Pract* 2018;72(3):e13070.
3. Björck S, Palaszewski B, Friberg L, et al. Atrial fibrillation, stroke risk, and warfarin therapy revisited: a population-based study. *Stroke* 2013;44(11):3103-08.
4. Katsanos AH, Kamel H, Healey JS, et al. Stroke prevention in atrial fibrillation: looking forward. *Circulation* 2020;142(24):2371-88.
5. Chao T-F, Nedeljkovic MA, Lip GY, et al. Stroke prevention in atrial fibrillation: comparison of recent international guidelines. *Eur Heart J Suppl* 2020;22(Supplement_O):O53-O60.
6. Xiong Q, Proietti M, Senoo K, et al. Asymptomatic versus symptomatic atrial fibrillation: a systematic review of age/gender differences and cardiovascular outcomes. *Int J Cardiol* 2015;191:172-77.
7. Freedman B, Camm J, Calkins H, et al. Screening for atrial fibrillation: a report of the AF-SCREEN International Collaboration. *Circulation* 2017;135(19):1851-67.
8. Hindricks G, Potpara T, Dagres N, et al. 2020 ESC Guidelines for the diagnosis and management of atrial fibrillation developed in collaboration with the European Association for Cardio-Thoracic Surgery (EACTS) The Task Force for the diagnosis and management of atrial fibrillation of the European Society of Cardiology (ESC) Developed with the special contribution of the European Heart Rhythm Association (EHRA) of the ESC. *Eur Heart J* 2021;42(5):373-498.
9. Lee J, Reyes BA, McManus DD, et al. Atrial fibrillation detection using an iPhone 4S. *IEEE Trans Biomed Eng* 2012;60(1):203-06.
10. Orchard J, Lowres N, Freedman SB, et al. Screening for atrial fibrillation during influenza vaccinations by primary care nurses using a smartphone electrocardiograph (iECG): a feasibility study. *Eur J Prev Cardiol* 2016;23(2_suppl):13-20.
11. Bowry AD, Lewey J, Dugani SB, et al. The burden of cardiovascular disease in low-and middle-income countries: epidemiology and management. *Can J Cardiol* 2015;31(9):1151-59.
12. Soni A, Karna S, Patel H, et al. Study protocol for Smartphone Monitoring for Atrial fibrillation in Real-Time in India (SMART-India): a community-based screening and referral programme. *BMJ Open* 2017;7(12):e017668.
13. Davidson KW, Barry MJ, Mangione CM, et al. Screening for atrial fibrillation: US Preventive Services Task Force recommendation statement. *JAMA* 2022;327(4):360-67.
14. Stavrakis S, Elkholey K, Lofgren MM, et al. Screening for atrial fibrillation in American Indian adults in a tribal primary care clinic. *J Am Heart Assoc* 2021;10(9):e020069.
15. Bai Y, Wang Y-L, Shantsila A, et al. The global burden of atrial fibrillation and stroke: a systematic review of the clinical epidemiology of atrial fibrillation in Asia. *Chest* 2017;152(4):810-20.
16. Rosengren A, Smyth A, Rangarajan S, et al. Socioeconomic status and risk of cardiovascular disease in 20 low-income, middle-income, and high-income countries: the Prospective Urban Rural Epidemiologic (PURE) study. *Lancet Glob Health* 2019;7(6):e748-e60.
17. Santos IS, Goulart AC, Olmos RD, et al. Atrial fibrillation in low-and middle-income countries: a narrative review. *Eur Heart J Suppl* 2020;22(Supplement_O):O61-O77.
18. Northern Provincial Council. Vital Statistics 2020. Jaffna, Sri Lanka: Office of the Deputy Chief Secretary, 2020.
19. Central Bank of Sri Lanka. Provincial Gross Domestic Product - 2019. Colombo, Sri Lanka: Statistics Department, 2020.
20. Patino CM, Ferreira JC. Inclusion and exclusion criteria in research studies: definitions and why they matter. *J Bras Pneumol* 2018;44:84-84.

For peer review only

21. O'Neill J, Tayebjee MH. Why are South Asians seemingly protected against the development of atrial fibrillation? A review of current evidence. *Trends Cardiovasc Med* 2017;27(4):249-57.
22. Chang T, Gajasinghe S, Arambepola C. Prevalence of stroke and its risk factors in urban Sri Lanka: population-based study. *Stroke* 2015;46(10):2965-68.
23. Martínez-Mesa J, González-Chica DA, Bastos JL, et al. Sample size: how many participants do I need in my research? *An Bras Dermatol* 2014;89:609-15.
24. Department of Census and Statistics Sri Lanka. Demographic and Health Survey 2016 Sri Lanka: Department of Census and Statistics Sri Lanka, 2016.
25. Austin SR, Wong Y-N, Uzzo RG, et al. Why summary comorbidity measures such as the Charlson comorbidity index and Elixhauser score work. *Med Care* 2015;53(9):e65.
26. Kroenke K, Spitzer RL, Williams JB, et al. Anxiety disorders in primary care: prevalence, impairment, comorbidity, and detection. *Ann Intern Med* 2007;146(5):317-25.
27. Arroll B, Goodyear-Smith F, Crengle S, et al. Validation of PHQ-2 and PHQ-9 to screen for major depression in the primary care population. *Ann Fam Med* 2010;8(4):348-53.
28. Lee PH, Macfarlane DJ, Lam TH, et al. Validity of the international physical activity questionnaire short form (IPAQ-SF): A systematic review. *Int J Behav Nutr Phys Act* 2011;8(1):1-11.
29. Bush K, Kivlahan DR, McDonell MB, et al. The AUDIT alcohol consumption questions (AUDIT-C): an effective brief screening test for problem drinking. *Arch Intern Med* 1998;158(16):1789-95.
30. World Health Organization. WHO STEPS surveillance manual: the WHO STEPwise approach to chronic disease risk factor surveillance. Geneva: World Health Organization, 2005.
31. Netzer NC, Stoohs RA, Netzer CM, et al. Using the Berlin Questionnaire to identify patients at risk for the sleep apnea syndrome. *Ann Intern Med* 1999;131(7):485-91.
32. Herdman M, Gudex C, Lloyd A, et al. Development and preliminary testing of the new five-level version of EQ-5D (EQ-5D-5L). *Qual Life Res* 2011;20(10):1727-36.
33. Revenig LM, Canter DJ, Kim S, et al. Report of a simplified frailty score predictive of short-term postoperative morbidity and mortality. *J Am Coll Surg* 2015;220(5):904-11. e1.
34. Schooling CM, Ho SY, Leung GM, et al. Diet synergies and mortality—a population-based case–control study of 32 462 Hong Kong Chinese older adults. *Int J Epidemiol* 2006;35(2):418-26.
35. Galloway CD, Albert DE, Freedman SB. iPhone ECG application for community screening to detect silent atrial fibrillation: a novel technology to prevent stroke. *Int J Cardiol* 2013;165:193-94.
36. Hall A, Mitchell ARJ, Wood L, et al. Effectiveness of a single lead AliveCor electrocardiogram application for the screening of atrial fibrillation: a systematic review. *Medicine (Baltimore)* 2020;99(30)
37. Cheung CC, Krahn AD, Andrade JG. The emerging role of wearable technologies in detection of arrhythmia. *Can J Cardiol* 2018;34(8):1083-87.
38. Beane A, De Silva AP, Athapattu PL, et al. Addressing the information deficit in global health: lessons from a digital acute care platform in Sri Lanka. *BMJ Glob Health* 2019;4(1):e001134.
39. REDCap. REDCap: Research Electronic Data Capture Nashville, Tennessee: Vanderbilt University; [Available from: https://projectredcap.org/.
40. Wilkinson MD, Dumontier M, Aalbersberg IJ, et al. The FAIR Guiding Principles for scientific data management and stewardship. *Sci Data* 2016;3(1):1-9.
41. CRIT Care Asia, Hashmi M, Beane A, et al. Leveraging a cloud-based critical care registry for COVID-19 pandemic surveillance and research in low-and middle-income countries. *JMIR Public Health Surveill* 2020;6(4):e21939.
42. Information and Communication Technology Agency of Sri Lanka. Security Sri Lanka: Information and Communication Technology Agency of Sri Lanka; [Available from: https://www.icta.lk/security/.

For peer review only

43. Centers for Disease Control Prevention. HIPAA privacy rule and public health. Guidance from CDC and the US Department of Health and Human Services. *MMWR Morb Mortal Wkly Rep* 2003;52(Suppl 1):1-17, 19.
44. Cook N, Siddiqi N, Twiddy M, et al. Patient and public involvement in health research in low and middle-income countries: a systematic review. *BMJ Open* 2019;9(5):e026514.
45. Arsenault C, Kim MK, Aryal A, et al. Hospital-provision of essential primary care in 56 countries: determinants and quality. *Bull World Health Organ* 2020;98(11):735.
46. Chow CK, Nguyen TN, Marschner S, et al. Availability and affordability of medicines and cardiovascular outcomes in 21 high-income, middle-income and low-income countries. *BMJ Glob Health* 2020;5(11):e002640.
47. Lowres N, Giskes K, Hespe C, et al. Reducing stroke risk in atrial fibrillation: adherence to guidelines has improved, but patient persistence with anticoagulant therapy remains suboptimal. *Korean Circ J* 2019;49(10):883-907.

For peer review only

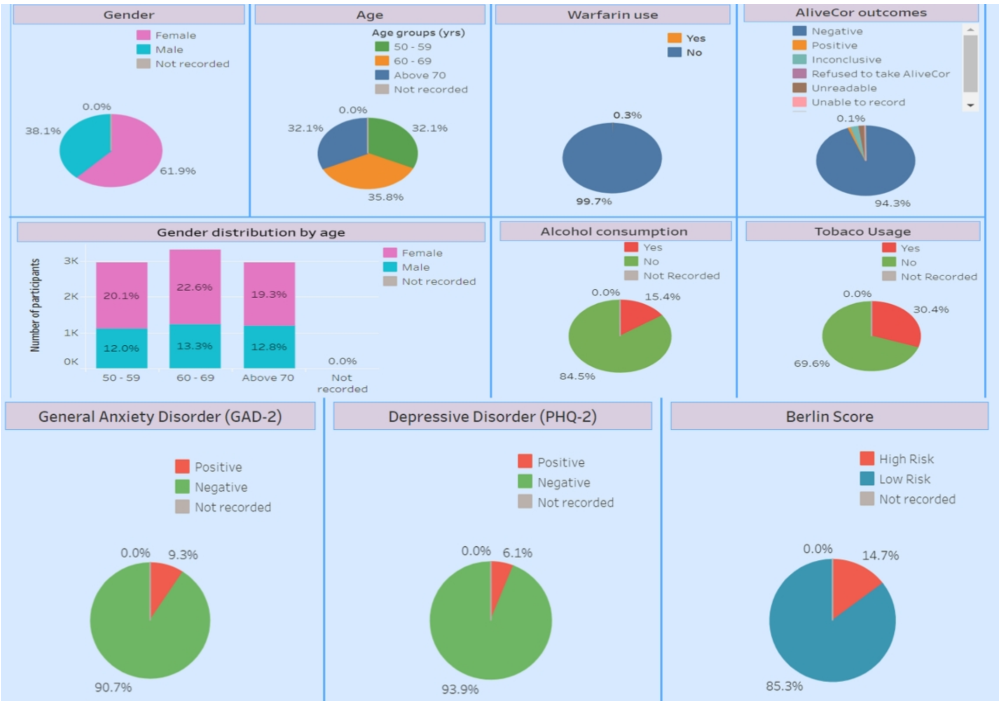

Analytical online dashboard. A breakdown of demographics, lifestyle and wellbeing measures for participants recruited and consented thus far.
